# Cryptic Binding Pockets in PDC‑3 β‑Lactamase Modulate Resistance Profiles

**DOI:** 10.1021/jacsau.5c01707

**Published:** 2026-02-20

**Authors:** Shuang Chen, Fedaa Attana, Andrea M. Hujer, Christopher R. Bethel, Magdalena A. Taracila, Robert A. Bonomo, Shozeb Haider

**Affiliations:** † 371646 UCL School of Pharmacy, London WC1N 1AX, U.K.; ‡ Research Service, Louis Stokes Cleveland Department of Veterans Affairs Medical Center, Cleveland, Ohio 44106-1702, United States; § Department of Molecular Biology and Microbiology, 12304 Case Western Reserve University School of Medicine, Cleveland, Ohio 44106-5029, United States; ∥ Department of Medicine, 12304 Case Western Reserve University School of Medicine, Cleveland, Ohio 44106-5029, United States; # Departments of Pharmacology, Biochemistry, and Proteomics and Bioinformatics, 12304 Case Western Reserve University School of Medicine, Cleveland, Ohio 44106-5029, United States; ∇ CWRU-Cleveland VAMC Center for Antimicrobial Resistance and Epidemiology (Case VA CARES), Cleveland, Ohio 44106-5029, United States; ○ Prince Fahd bin Sultan Chair for Biomedical Research (PFSCBR), University of Tabuk, Tabuk 71491, Saudi Arabia

**Keywords:** β-lactamase, cryptic pocket, antibiotic resistance, molecular dynamics, deep learning

## Abstract

Cryptic binding pockets in proteins can modulate catalysis, allostery, and druggability. Yet they are rarely captured by experiments or conventional molecular dynamics simulations. Here, we combine enhanced sampling with an unsupervised deep-learning pipeline to map the full conformational landscape of the Ω-loop in class C β-lactamase PDC-3. Three principal conformational ensembles were identified: a crystal-like state resembling the native structure, an expansive state characterized by widening of the active-site cleft, and a constricted state that blocks access to the catalytic site. Residues 219 and 221 act as molecular switches that shuffle the enzyme between these states and thereby modulate the resistance profiles. Steady-state inhibition assays with nitrocefin and bulky cephalosporins confirm that substitutions at these positions selectively reshaped the binding pocket. In addition, across multiple expansive states, D217 repeatedly forms a salt bridge with K67 in a geometry reminiscent of general base E166 of class A enzymes. Thus, it is plausible that D217 might transiently adopt a ‘backup’ general base role under certain conformational states. Most strikingly, occlusion of the catalytic site reveals a previously unseen cryptic pocket, offering an attractive allosteric target for inhibitors that would lock PDC-3 in a catalytically incompetent conformation. The integrated framework proposed in this study is highly generalizable and can serve as a powerful tool for identifying hidden protein conformations and uncovering previously inaccessible regulatory mechanisms.

## Graphical abstract

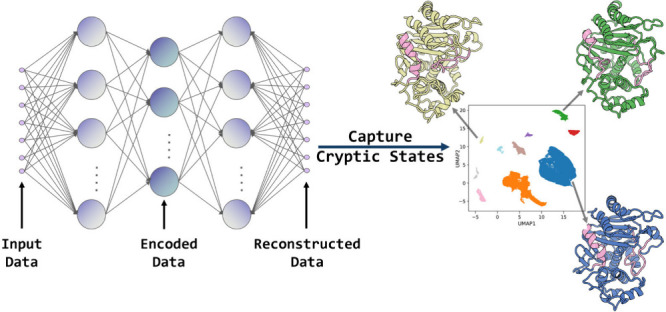

## Introduction

Proteins exist as dynamic ensembles of conformations, within which transient hidden or cryptic states can emerge through intrinsic motions.^1,2^ These hidden states, including buried pockets or alternative loop arrangements that are not captured by static crystal structures, can hold significant functional and pharmacological relevance.^2^ Accumulating evidence indicates that enzymes leverage conformational flexibility to regulate activity, enable allostery, and even reveal binding sites for inhibitors.^3,4^ However, detecting cryptic pockets or rare conformational states is challenging, as they occur infrequently and may be missed by conventional experiments.^1,2^ Classical molecular dynamics simulations can in principle reveal cryptic sites, but exhaustive sampling of rugged energy landscapes is computationally expensive and the resulting high-dimensional data are difficult to interpret.^5^ As a result, it often remains unclear whether these states simply represent insignificant fluctuations or confer a beneficial functional advantage.^6^

A clear illustration of this challenge arises in the study of β-lactamases, where transient loop rearrangements can dictate antibiotic resistance. In particular, class C β-lactamase PDC-3 (*Pseudomonas*-derived cephalosporinase) provides a compelling case. PDC-3 is a serine β-lactamase capable of hydrolyzing a broad range of β-lactam antibiotics.^7,8^ Notably, the Ω-loop of PDC-3 is a highly flexible and mutational hotspot, with residues such as V211, G214, E219, and Y221 frequently undergo substitutions.^9,10^ All residues in this study are numbered based on the Structural alignment-based numbering of class C β-lactamase scheme (SANC).^11^ These mutations have been found to significantly enhance antibiotic resistance by altering enzyme dynamics and substrate specificity.^9^ Variants like E219 K and Y221H have been observed to confer increased resistance to ceftolozane and ceftazidime, limiting therapeutic options.^9,12^ Previous studies have proposed that these mutations alter the conformational landscape of the Ω-loop, potentially enlarging the active site or reconfiguring key hydrogen bonds within the active-site network.^9,10^ Despite these insights, comprehensive studies specifically characterizing the full spectrum of Ω-loop conformational transitions remain lacking.

Given the critical role of Ω-loop mutations in conferring antibiotic resistance, it is essential to investigate how these mutations affect the structural and functional dynamics of PDC-3. The present work focuses on PDC-3 Ω-loop as a model system to develop an integrated strategy for capturing and analyzing hidden conformational states. The Ω-loop in PDC-3 provides an excellent testbed as it is inherently flexible, its mutations are clinically important, and prior evidence hints at cryptic loop-expansion states that modulate antibiotic resistance.^8,10^ Traditional simulation approaches (e.g., long classical MD) may provide only a rare glimpse of these states. Therefore, we employ well-tempered metadynamics (WT-MetaD), an enhanced sampling technique, to drive transitions in the Ω-loop conformation (Figure 1). By using appropriate collective variables, WT-MetaD can flatten energy barriers and reveal multiple metastable basins within feasible simulation time.^13^ Compared with other enhanced-sampling strategies such as accelerated MD (aMD/GaMD),^14^ replica-exchange methods,^15^ and steered MD,^16^ WT-MetaD explicitly defined collective variables (CVs) represent the motions of interest. This makes the acceleration mechanistically interpretable and directly controllable, rather than applying a global acceleration that can introduce nonspecific effects. However, capturing the states has only partially solved the problem; making sense of the vast simulation data set is another challenge. To this end, we leverage unsupervised deep learning in the form of a convolutional variational autoencoder (CVAE) trained on protein conformations. Variational autoencoders are capable of learning low-dimensional representations of complex structural ensembles.^17^ Here, we use CVAE to encode protein distance-matrix data into a latent feature space that should reflect the major conformational degrees of freedom of the Ω-loop. By further applying dimensionality reduction (Uniform Manifold Approximation and Projection, UMAP) and clustering (Hierarchical Density-Based Spatial Clustering of Applications with Noise, HDBSCAN) on the CVAE latent space, we can detect distinct clusters corresponding to different conformational states.^18–20^ We then analyze the resulting clusters to identify structural rearrangements that explain how each mutation influences the ensemble. By integrating enhanced sampling methods with an unsupervised deep-learning pipeline to investigate the elusive conformational states of PDC-3 and its variants, we (i) demonstrate a broadly applicable strategy to capture cryptic protein conformations that remain inaccessible to crystallography and classical molecular dynamics simulations; (ii) establish a mechanistic overview across distinct β-lactamase classes, providing a unified perspective on the evolution of antibiotic resistance; and (iii) open new avenues for the rational design of next-generation β-lactam antibiotics and β-lactamase inhibitors.

**Figure 1:**
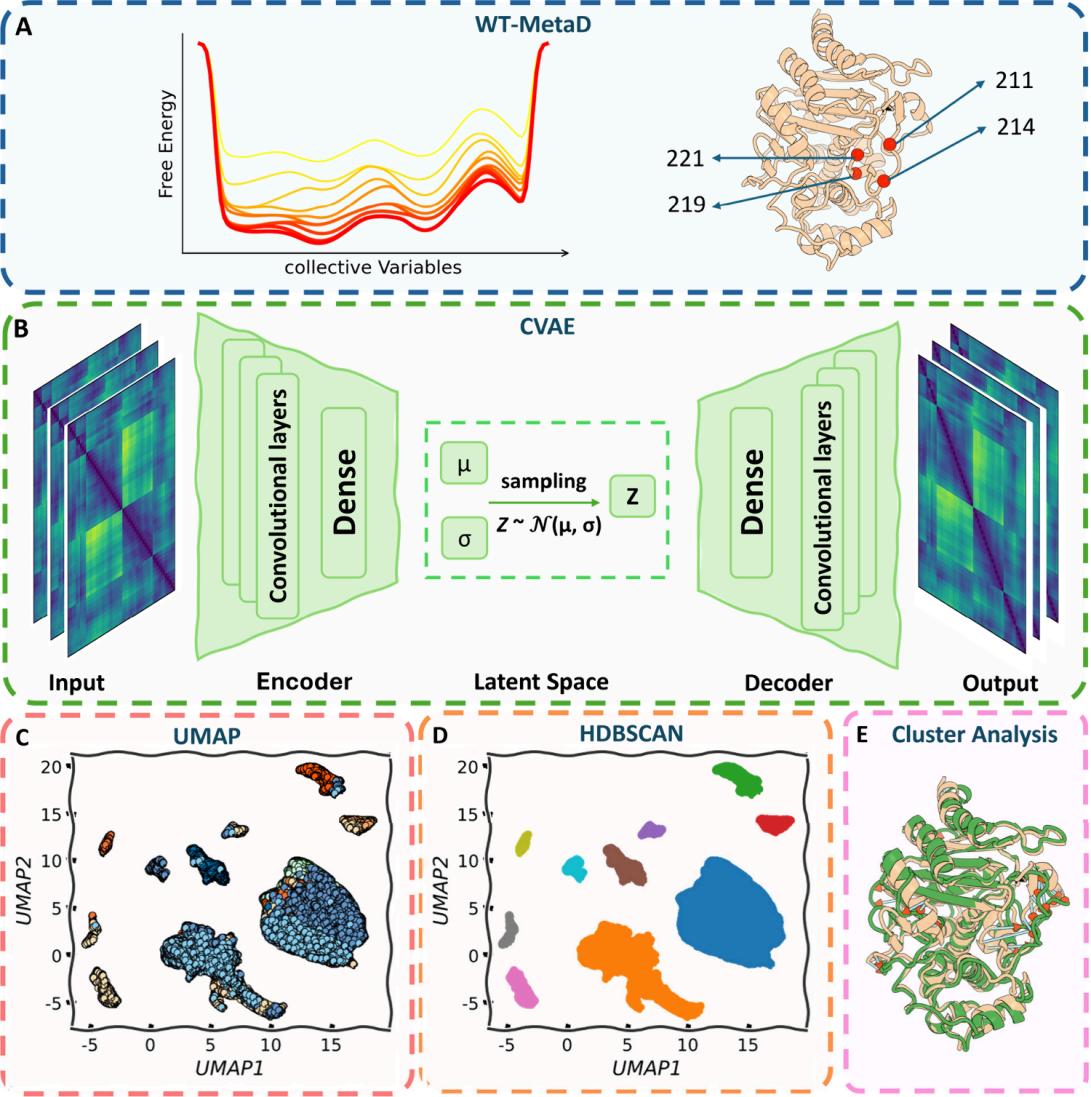
Schematic overview of the integrative computational strategy. (A) Well-tempered metadynamics (WT-MetaD) simulations bias backbone ϕ/ψ angles at critical Ω-loop residues (V211, G214, E219, Y221) in wild-type PDC-3 and its mutants, generating extensive ensembles of low-free-energy conformations. (B) Each low-energy frame is encoded as a distance matrix that captures structural details of catalytic motifs, the Ω-loop, helix 10, and nearby active-site residues and then processed using a convolutional variational autoencoder (CVAE). (C) CVAE’s resulting 8-dimensional latent embeddings are projected into two dimensions via Uniform Manifold Approximation and Projection (UMAP). (D) The 2D projections are subsequently clustered with Hierarchical Density-Based Spatial Clustering of Applications with Noise (HDBSCAN) to partition conformations into discrete structural basins. (E) Each identified cluster undergoes detailed structural characterization to elucidate how specific mutations influence Ω-loop expansion or constriction, thereby reshaping the active-site network.

## Results and Discussion

### Enhanced Sampling Captures Significant Conformational Changes Induced by Residues 219 and 221

To explore how variations at residues V211, G214, E219, and Y221 influence the dynamic behavior of PDC-3, we performed well-tempered metadynamics simulations using their backbone dihedral torsion angles as key collective variables (CVs) (Figures S1–S13). From these simulations, we selected frames exhibiting stable conformations defined by free energies below – 200 kJ/mol for detailed structural analysis to reduce the impact of short-lived bias-driven distortions (Figures S14 and Table S1). From these low-energy frames, we quantified the global and local structural effects through root-mean-square deviation (RMSD), root-mean-square fluctuation (RMSF), and side chain χ_1_ angle analyses (Figures S15–S20).

When backbone torsions at V211 or G214 were biased in wild-type PDC-3, the enzyme’s conformation remained similar to the crystalline form. The RMSD values rarely exceed 2.0 Å, and any rise in the RMSF values was confined to peripheral segments of the Ω-loop (Figure 2A, B and C). Conversely, E219 and Y221 serve as potent switches. Biasing E219 or Y221 in wild-type PDC-3 often drove the RMSD above 2.5 Å. This markedly amplified the RMSF around the catalytic pocket, indicating substantial reorganization of the active-site landscape. These findings suggest that the backbone torsion changes of PDC-3 residues 211 and 214 do not significantly alter the conformation of the active site, while residues 219 and 221 have the capacity to reorganize loop regions in a manner that could reshape the active-site architecture.

**Figure 2:**
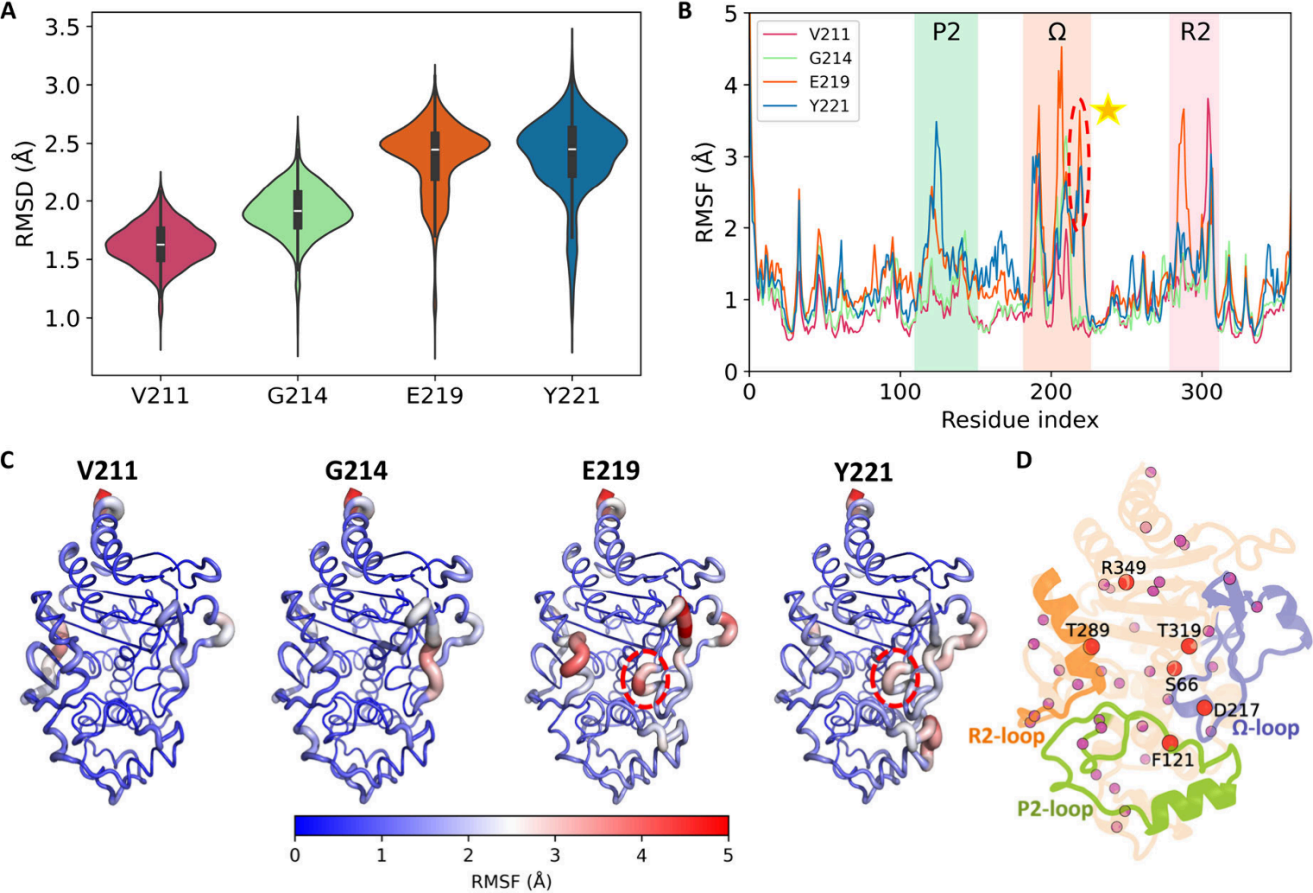
Global and local conformational changes induced by backbone-torsion biasing at V211, G214, E219, and Y221 of wild-type PDC-3. (A) Violin plots of RMSD distributions (in Å) for low-free-energy frames (free energy < –200 kJ/mol) obtained when backbone ψ/ϕ torsions at V211 (red), G214 (green), E219 (orange), or Y221 (blue) are biased. The white line indicates the median, and the black box denotes the interquartile range. (B) Per-residue RMSF profiles (in Å) for each torsion-biasing condition: V211 (red), G214 (green), E219 (orange), Y221 (blue). Shaded regions mark the P2-, Ω-, and R2-loops. The dashed red circle highlights the prominent RMSF peaks in the Ω-loop when E219 or Y221 is biased. (C) Putty representations of PDC-3 under each torsion bias (V211, G214, E219, or Y221), colored from blue→white→red according to per-residue RMSF and displayed with tube thickness proportional to RMSF magnitude. The dashed red circle highlights dynamic region in the Ω-loop near the catalytic residues under E219 or Y221 bias. (D) Mapping of 40 residues onto the PDC-3 structure whose χ_1_ distributions show Jensen-Shannon divergence > 0.5 relative to the wild-type PDC-3 (V211 reference), with six residues in the active site, Ω-, P2 and R2-loops specifically highlighted.

Examining mutant systems reinforced these structural insights (Figure S15). Specifically, the V211A substitution largely preserved the enzyme’s native structure, whereas V211G induced minor increases in local mobility within both the Ω- and R2-loops but did not provoke substantial active-site restructuring. Similarly, G214A induced moderate structural fluctuations in the active-site-proximal Ω-loop, with RMSF peaks reaching around 4 Å. Likewise, the G214R substitution predominantly affected the peripheral regions of the Ω-loop and R2-loop. In contrast, E219 and Y221 substitutions trigger major conformational changes and have the most dramatic effects on antibiotic resistance. Biasing these backbone positions in simulations drove large RMSD shifts (>2.5 Å) and greatly increased loop flexibility, signaling an active-site reorganization. Experimentally, substitutions at 219 or 221 are well-known to expand AmpC β-lactamase spectra.^9,10,12^ These structural perturbations were strongly associated with dramatic increases in resistance, particularly against large-side chain cephalosporins such as ceftolozane and ceftazidime, with MIC enhancements ranging from 8-fold to 128-fold, substantially exceeding those induced by mutations at V211 or G214.^9^ In addition, the active-site-proximal Ω-loop region showed extremely high RMSF peaks, highlighting how specific side chain changes at these sites can further magnify torsion-induced transitions. Variants like E219 K (around 10 Å) and Y221A (around 6 Å) stood out in driving large-scale fluctuations, consistent with previous studies.^10^ The E219 K boosted ceftazidime MIC from ∼2 to 64 μg/mL (32-fold) and ceftolozane MIC from ∼0.5 to 64 μg/mL (128-fold), while Y221A elevated ceftolozane MIC 64-fold (0.5 → 32 mg/L) and ceftazidime MIC 16-fold (2 → 32 mg/L).^9^ The extremely high RMSF values, together with the dramatic increase in resistance, indicate that E219 K and Y221A may unlock a hidden capacity to hydrolyze drugs that wild-type AmpC could not.

To gain additional insight into how these backbone torsion changes propagate through PDC-3, we computed side chain χ_1_ torsion angles for each residue across all simulated systems. The wild-type V211 system, which closely mirrors the crystal structure, as evidenced by consistently low RMSD values, served as the reference. Forty residues exhibited Jensen-Shannon (JS) divergence values exceeding 0.5 compared to the V211 reference, marking them as sites with notably altered side chain conformations (Figure 2D). Among these residues, only six residues, including S66, F121, D217, T289, T319, and R349 lie within or near the active-site pocket (Figures S20 and S21, Table 1 and Table S2).

**Table 1:** Jensen-Shannon (JS) Divergence of Side Chain *χ*_1_ Distributions for Six Active-Site Residues, Referenced to the Wild-Type PDC-3 (V211 System)^a^

	V211	V211A	V211G	G214	G214A	G214R	E219	E219A	E219G	E219 K	Y221	Y221A	Y221H
S66	0.00	0.17	0.06	0.35	**0.59**	**0.74**	0.46	**0.58**	0.41	**0.60**	**0.64**	0.34	**0.52**
F121	0.00	0.00	0.00	0.03	0.11	0.13	0.01	0.20	0.25	**0.58**	0.04	0.19	0.13
D217	0.00	0.00	0.00	0.01	0.19	0.21	**0.84**	0.48	**0.79**	**0.89**	**0.83**	**0.50**	**0.85**
T289	0.00	0.03	0.01	0.01	0.00	**0.80**	0.00	**0.74**	**0.75**	**0.72**	0.00	**0.70**	**0.72**
T319	0.00	0.04	0.01	0.11	0.01	0.42	0.05	**0.55**	**0.62**	**0.52**	0.35	0.49	**0.73**
R349	0.00	0.43	0.44	0.43	0.42	**0.52**	0.34	**0.52**	**0.51**	**0.52**	0.46	**0.51**	**0.52**

a The table lists the JS divergence between each variant and wild-type PDC-3 with a backbone bias at V211 (reference). A value of 0 denotes unchanged rotamer preferences, whereas values approaching 1 indicate a substantial redistribution of *χ*_1_ populations. Divergences equal to or exceeding 0.50 are taken as the threshold for a significant conformational shift and are highlighted in bold.

The V211 variants and wild-type G214 systems typically did not surpass the 0.5 JS threshold; however, mutations at residue 214 (G214A and G214R) significantly shifted S66’s χ_1_ angle, indicating a direct transmission of structural perturbations to the catalytic motif S64–K67. Similarly, pronounced alterations at S66 were consistently observed in variants E219A, E219 K, Y221A, and Y221H, indicating that mutations at residues 219 and 221 robustly propagate structural changes to the catalytic site. Furthermore, substitutions at residues E219 and Y221 significantly affected T319, a residue from another conserved catalytic motif, highlighting their extensive structural and functional impact. Residue F121 exhibited a JS divergence above 0.5 only in the E219 K mutant, suggesting that E219 K has a particularly strong effect on the P2-loop conformation. Residue T289, positioned within the R2 loop, remained largely unaffected by wild-type movements of V211, G214, E219, or Y221, yet showed a JS divergence of 0.8 in G214R and similarly high values in multiple E219 and Y221 mutants. In parallel, these same variants also affect R349, a residue essential for substrate binding at the active site and positioned adjacent to the R2-loop.^21^ Such findings indicate that E219, Y221 mutants, and G214R can substantially influence the R2 binding site responsible for accommodating the R2 side chain of cephalosporin antibiotics. Residue D217, located within the active-site-proximal Ω-loop region, uniquely demonstrated negligible conformational shifts upon alterations at residues 211 and 214 but exhibited pronounced changes under both wild-type and mutant conditions involving residues 219 and 221. This observation, consistent with RMSD/RMSF analyses, reinforces the role of residues 219 and 221 as pivotal switches capable of propagating torsional rearrangements across the enzyme’s structural network.

To test these simulation-based predictions, we investigated how these Ω-loop substitutions affect enzyme function. We performed steady-state inhibition assays in which nitrocefin (NCF) served as a chromogenic reporting substrate, and ceftazidime and ceftolozane were added as competing ligands. Although they are hydrolyzable substrates rather than classical inhibitors, under our conditions their turnover is slow compared with nitrocefin, so they effectively behave as competitive inhibitors of NCF hydrolysis. NCF kinetics remain remarkably robust across all variants (Table 2), i.e., within roughly 2-fold of wild-type PDC-3. This indicates that these mutations do not disrupt the conserved catalytic core and do not grossly impair generic β-lactamase activity toward the relatively compact reporter substrate nitrocefin, whose small scaffold makes only limited steric demands on the active site. By contrast, ceftazidime and ceftolozane are much bulkier cephalosporins with extended side chains that probe the shape and dynamics of the Ω-loop and R2-loop far more stringently. Accordingly, the apparent inhibition constants for ceftazidime and ceftolozane clearly distinguish variants carrying mutations at V211/G214 from those with substitutions at E219/Y221 (Figures S22 and S23). Substitutions at V211 and G214 produce only modest changes in the apparent inhibition constants for ceftazidime (0.5–2 fold) and ceftolozane (3–4 fold), consistent with their localized loop flexibility and the moderate MIC increases previously reported for these variants.^9^ In contrast, substitutions at E219 and Y221, which our simulations identify as molecular switches, lead to much larger increases in *K*_i_, ranging from 4- to 50-fold for ceftazidime and from 5- to 7-fold for ceftolozane. The pattern and magnitude of these changes closely mirror earlier MIC data,^9^ supporting the conclusion that mutations at positions 219 and 221 reorganize Ω-loop and R2-loop geometry in a way that strongly reshapes the binding pocket for bulky cephalosporins, whereas mutations at 211 and 214 exert much weaker effects.

**Table 2:** Steady-State Kinetic Parameters for Nitrocefin (*V*_max,NCF_ and *K*_m,NCF_), Apparent Competitive Inhibition Constants for Ceftazidime and Ceftolozane (*K*_i__,CAZ_ and *K*_i__,TOL_) for Wild-Type PDC-3 and Its Variants^a^

	*V*_max,NCF_	*K*_m,NCF_ (μM)	*K*_i,CAZ_ (μM)	*K*_i,TOL_ (μM)
PDC-3	0.94 ± 0.12	36 ± 12	55 ± 9	77 ± 26
V211A	1.30 ± 0.10	24 ± 6	26 ± 7	272 ± 25
V211G	1.07 ± 0.06	26 ± 5	40 ± 8	317 ± 49
G214A	1.07 ± 0.17	13 ± 9	101 ± 14	301 ± 35
G214R	1.15 ± 0.12	20 ± 8	118 ± 25	237 ± 41
E219A	1.28 ± 0.05	28 ± 3	303 ± 55	538 ± 34
E219G	1.03 ± 0.19	29 ± 15	239 ± 32	397 ± 58
E219 K	1.25 ± 0.11	22 ± 7	398 ± 48	403 ± 40
Y221A	0.96 ± 0.07	24 ± 6	482 ± 39	408 ± 33
Y221H	1.05 ± 0.02	21 ± 2	2700 ± 300	458 ± 52

a *K*_i_ values are corrected for nitrocefin turnover under the assay conditions and report the apparent affinity of ceftazidime or ceftolozane for the nitrocefin-occupied active site.

### Unsupervised Clustering Differentiates Distinct Multiple Conformations

To systematically investigate the conformational states accessed in our simulations, we applied unsupervised deep learning and clustering to the ensemble of low-energy structures. The CVAE was trained on distance-matrix representations of the structures and then was used to embed each frame into a latent space. Visualization of the latent embeddings via UMAP and subsequent HDBSCAN clustering revealed ten distinct clusters (Figure 3A, B; see Figure S24 for CVAE training performance and Figure S25 for reconstruction quality). These clusters capture a range of Ω-loop conformations in PDC-3 and its variants: a canonical crystal-like ensemble (cluster 1; Figure 3C, D) in which closely resembles the experimentally determined crystal structure, an expansive ensemble (clusters 2–6; Figure 4) in which the Ω-loop has swung outward and the active-site cleft is widened, and a constricted ensemble (clusters 7–10; Figure 5) in which the Ω-loop has shifted inward narrowing or occluding the active site (Figure S26, Tables S3 and S4).

**Figure 3:**
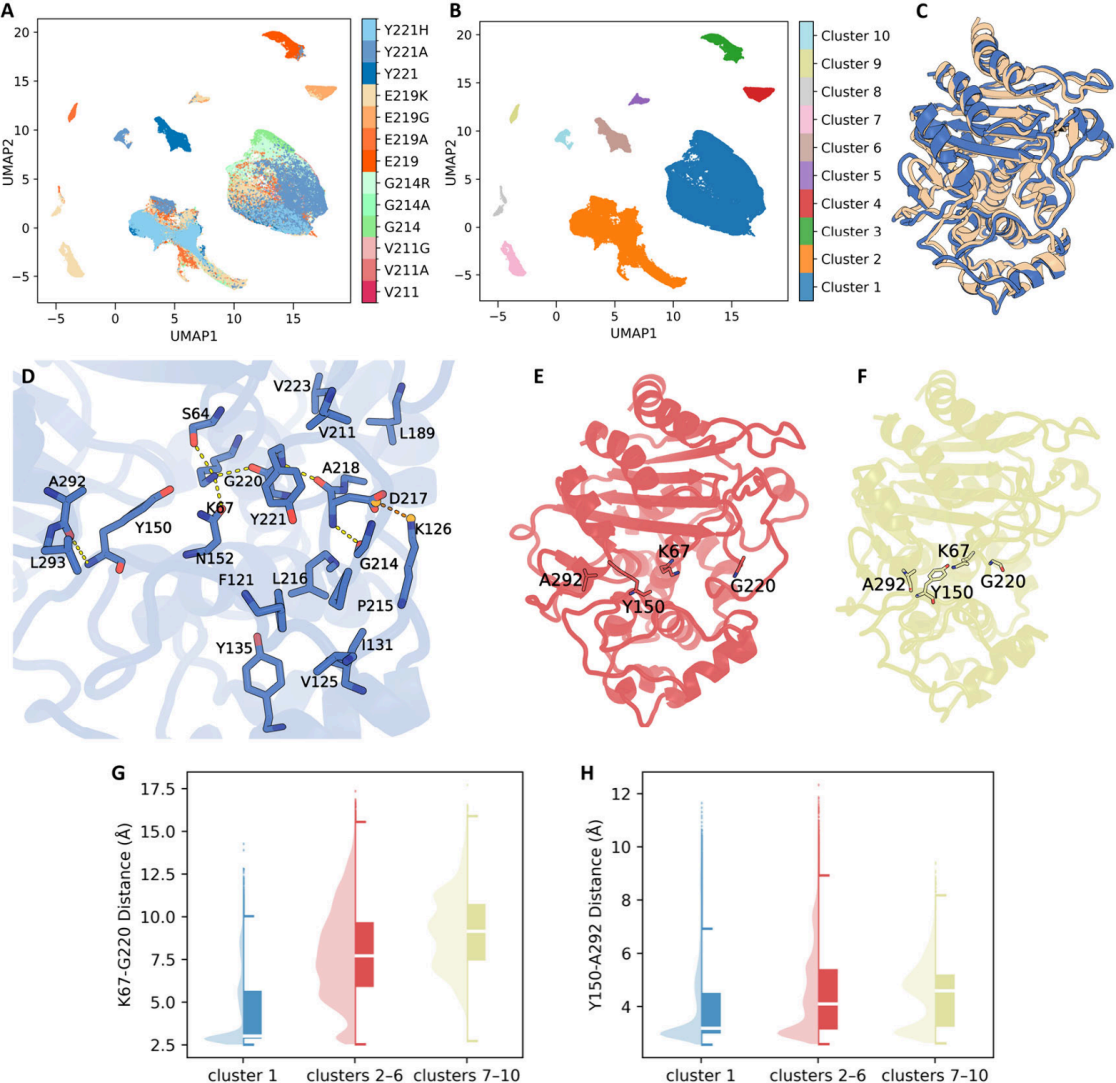
CVAE-UMAP clustering reveals distinct Ω-loop conformational states. (A) 2D UMAP projection of CVAE latent embeddings for all low-energy frames, colored by system identity. Each point corresponds to a single frame sampled from residue 211, 214, 219, and 221 torsion biases (including wild-type and mutants). (B) The same UMAP projection colored by HDBSCAN cluster assignment (clusters 1–10). (C) Structural alignment of a representative structure from cluster 1 (blue) onto the crystal structure (peach). (D) Detailed view of crystal-like (cluster 1) interactions. (E, F) Ribbon depictions of two representative conformations in which both K67–G220 and Y150–A292 hydrogen bonds are disrupted, but the Ω-loop displacement differs. In (E) (clusters 2–6, red), the Ω-loop swings outward, dramatically widening the active-site cleft. In (F) (clusters 7–10, lime), the Ω-loop instead shifts toward the R2 loop. (G–H) Violin-box plots of donor–acceptor distances for hydrogen bonds (G) K67–G220 and (H) Y150–A292 in crystal-like (cluster 1, blue), expansive (clusters 2–6, red), and constricted (clusters 7–10, lime) states. Each plot shows the full distribution (violin), median and interquartile range (box), whiskers at 1.5× IQR, and outliers as individual points.

**Figure 4:**
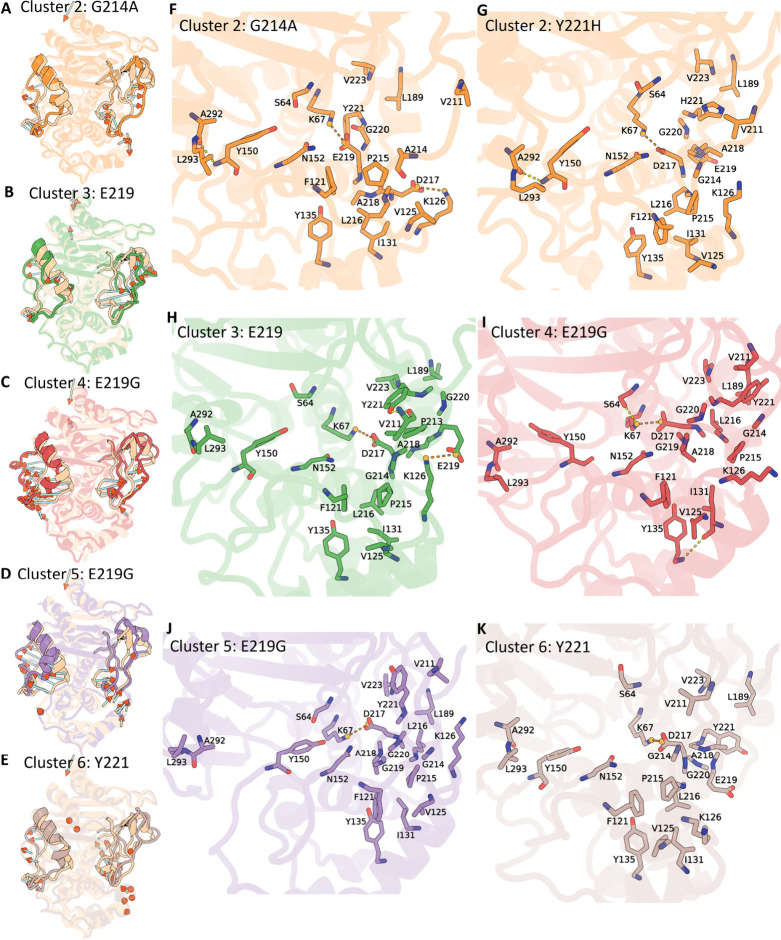
Expansive-state structural ensemble (clusters 2–6). (A–E) Representative snapshot structures from clusters 2–6, each aligned to the wild-type crystal reference (peach). The Ω-loop and R2-loop are highlighted, and arrows indicate Cα displacements exceeding 4 Å relative to the crystal. (F–K) Close-up interaction maps for those same conformations, with key residues shown in stick representation; hydrogen bonds are colored yellow and salt bridges orange.

**Figure 5:**
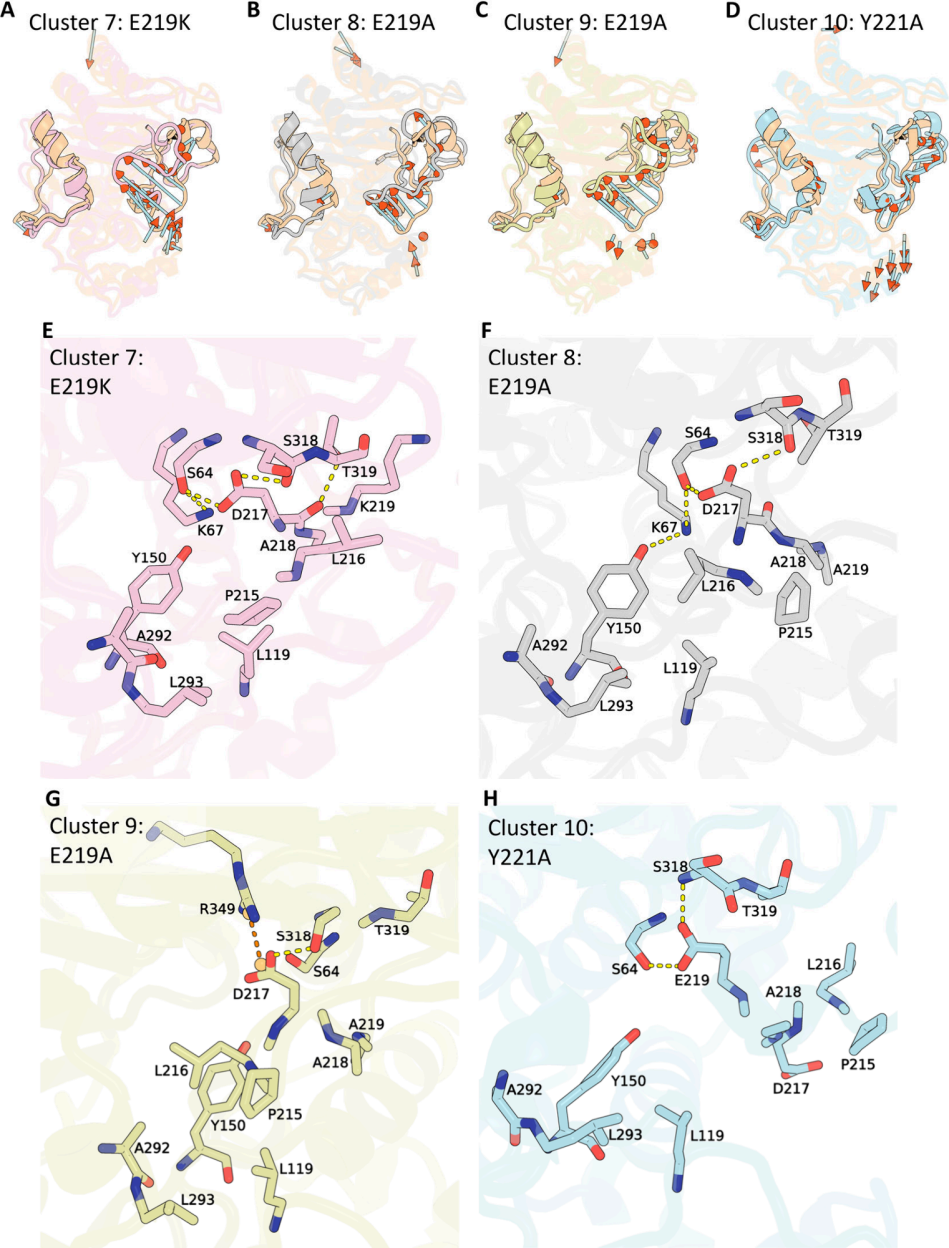
Constricted-state structural ensemble (clusters 7–10). (A–D) Representative structures from clusters 7–10, each aligned to the wild-type crystal reference (peach). The Ω-loop and R2-loop are highlighted, and arrows indicate Cα displacements exceeding 4 Å relative to the crystal. (E–H) Close-up interaction maps for those same conformations, with key residues shown in stick representation; Hydrogen bonds are colored yellow and salt bridges orange.

All systems could sample cluster 1 state to some extent, reflecting the intrinsic stability of the native conformation. When the backbone torsions of residue 211 or 214 in wild-type PDC-3 or residue 211 in V211 variants are biased, almost all conformations fall into crystal-like cluster 1. This behavior is consistent with the kinetic data, which show that V211 substitutions cause only modest changes relative to wild-type PDC-3 (Table 2). In contrast, when residue 214 is biased in the G214 variants, a substantial fraction of the ensemble populates expansive clusters outside the crystal-like basin, particularly for G214A. This redistribution of population toward expansive states provides a structural rationale for the ≈3-fold lower *K*_m_ of G214A compared with wild-type PDC-3 (36.3 ± 12.0 → 12.8 ± 8.9 μM). Biasing residues 219 or 221 even produces a much more diverse conformational distribution, with most frames falling outside the crystal-like cluster 1 into expansive or collapsed clusters. Thus, the cluster-population analysis supports our interpretation of residue 219 and 221 as molecular switches. Mutations at these positions redistribute population away from the crystal-like basin into alternative conformations that strongly remodel the binding pocket for bulky cephalosporins, in line with their pronounced kinetic and resistance profiles. Notably, E219 K sampled the broadest range of states. This high conformational flexibility of E219 K likely contributes to its enhanced catalytic activity and may help explain the superior catalytic performance observed for this variant. Moreover, it is important to note that relative population measures only as a qualitative check that the conformations we report are not isolated or accidental events, but recur and persist within the sampled ensemble, and they should not be interpreted as equilibrium populations.

Each cluster was examined in detail to clarify the specific structural features underlying these states (Supplementary Data 1 and Supplementary Note 1). In crystallike cluster 1, the Ω-loop remains snug against the active-site pocket, stabilized by an extensive interaction network that mirrors the crystal structure (Figure 3C and D). D217 appears crucial to this network. Centrally positioned within the Ω-loop and adjacent to the active site, D217 forms critical interactions with nearby backbone atoms of residues G220, Y221, or G222. Additionally, its backbone atoms establish robust hydrogen bonds with G214, further anchoring the Ω-loop in a configuration tightly wrapped around the catalytic pocket. The side chain of D217 also engages in a strong salt bridge with K126 on the P2-loop, significantly reinforcing the structural rigidity of the Ω-loop and restricting excessive flexibility. The importance of residue D217 is emphasized by its extraordinary conservation, as revealed by the additional analysis of 6688 class C β-lactamase sequences (Supplementary Data 2 and Supplementary Note 2). Among these sequences, 97.16% possess an aspartate at this position 217, strongly suggesting that evolutionary pressure maintains this residue due to its critical functional role. Additionally, hydrophobic interactions further stabilize the Ω-loop. The P215 interacts strongly with F121, V125, and I131, while L216 similarly engages F121, I131, and Y135. The hydrophobic contacts between V211–Y221 and A218/V223-L189 further maintain the conformation of the Ω-loop. These combined interactions firmly secure the Ω-loop near the catalytic pocket, preserving the crucial hydrogen bond between G220 and the catalytic residue K67. Furthermore, residue Y150 can form hydrogen bonds and hydrophobic interactions with residues A292 and L293 on the R2 loop. Collectively, the hydrogen bonds K67-G220 and Y150-A292 control the expansion and constriction of the active site, consistent with our earlier findings.^10^ The disruption of these specific bonds is a hallmark of the alternative states in clusters 2–10 (Figure 3E,H and Table S5).

Clusters 2–6 comprise the ensemble, which is characterized by a dramatic expansion of the active site. In these states, the loop moves outward (in some cases by over 10 Å displacement of the G220 Cα; Figure S27), enlarging the active-site cleft. The K67-G220 hydrogen bond is lost in almost all expansive-state frames, reflected by a substantial increase in the distance between residues K67 and G220 compared with the crystal state (Figure 3E, G and Figure S28). Similarly, the Y150-A292 interaction is weakened or broken, as the movement of the Ω-loop often couples with a slight displacement of the R2 loop (Figure 3F, H and Figure S28). Within this broad class of expansive conformations, our clustering distinguishes several substates with subtle differences (Figure 4). Cluster 2, for example, is populated predominantly by frames from the G214A and Y221H simulations. Specifically, they exhibit pronounced twisting of the Ω-loop carrying G220 far from K67, while preserving its internal contacts between P215/L216 and the P2-loop (Figure 4A, F, and G). Residue A218, which originally interacts hydrophobically with L189, moves considerably away. Residue 221 (Y221 in G214A and H221 in Y221H) reorients significantly, becoming perpendicular to the plane of the catalytic residues, thereby alleviating prior steric hindrance and enhancing ligand accessibility. Additionally, for variant G214A, the E219 side chain flips inward and forms a new salt bridge with K67. In the Y221H system, D217 rather than E219 plays a similar role, approaching K67 to establish a salt bridge. Cluster 3 represents another expansive state. Specifically, backbone torsion changes at residue E219 allow it to form a salt bridge with K126 (Figure 4B and H). Meanwhile, residue Y221 can establish hydrogen bonds with V211 and hydrophobic contacts with P213, which help stabilize the loop in its expansive position. In the E219G variants (clusters 4 and 5), significant backbone torsion shifts at residue 219 cause the Ω-loop to rotate, repositioning L216 from inward-facing orientations toward the active site to outward-facing positions and weakening the original hydrophobic interactions observed in clusters 1–3 (Figure 4C, D, I and J). These interactions are replaced by new hydrophobic contacts formed between A218 and residues F121/I131 on the P2-loop. Moreover, residue Y221 no longer points toward the catalytic pocket, thus enlarging the active site. Simultaneously, a notable displacement of the R2 loop disrupts the Y150-A292 interaction, greatly expanding the catalytic site. Additionally, backbone torsion changes in residue Y221 of wild-type PDC-3 (cluster 6) redirect Y221 outward from the catalytic pocket, markedly expanding the region near the catalytic residues (Figure 4E and K).

Notably, a prominent structural feature observed consistently across clusters 3–6 is the reinforced K67–D217 salt bridge, initially identified in cluster 2. Structural alignment of representative conformations with the crystal structure of KPC-2, a class A β-lactamase, revealed that residue D217 in PDC-3 occupies a position analogous to that of residue E166 in KPC-2 (Figure 6A). E166 is known to function as a general base, playing a pivotal role in the catalytic mechanism of class A β-lactamases.^22^ This observation prompts the question: could D217 assume a similar catalytic role under some conditions? Early work argued that class C β-lactamases lack an acidic residue equivalent to E166 in class A β-lactamases.^23^ Consistent with this view, comprehensive scanning mutagenesis of the P99 enzyme found the D217 variant to be functionally neutral, retaining ≈74% of wild-type *k*_cat_/*K*_m_.^24^ Furthermore, a recent microbiological data show that the D217 mutation in PDC AmpC β-lactamase even significantly increased MIC values for several cephalosporins.^25^ Yet, previous investigations have largely focused on cephalosporins, with fewer studies addressing penicillin or carbapenem substrates. Relative to PDC-5, the single D217N substitution in PDC-315 boosts catalytic efficiency against third-generation cephalosporins (e.g., *k*_cat_/*K*_m_ rises from 0.037 to 0.356 mM^–1^ s^–1^ for ceftolozane and from 0.151 to 0.240 mM^–1^ s^–1^ for ceftazidime) yet diminishes efficiency toward penicillins or carbapenems (e.g., piperacillin 127 → 39 mM^–1^ s^–1^; imipenem 9.0 → 2.1 mM^–1^ s^–1^). Corresponding MICs in PAOΔC strains mirror these kinetic shifts, and competitive IC50 assays reveal a > 300-fold loss of affinity for the penicillin scaffold cloxacillin while avibactam binding remains unchanged.^26^ As mentioned earlier, D217 significantly contributes to stabilizing the Ω-loop and active-site architecture. Mutation at this position likely enhances Ω-loop flexibility, expanding the pocket to accommodate cephalosporins with bulky side chains and, thereby, enhancing resistance. Conversely, this structural shift appears to be detrimental to penicillins or carbapenems, potentially implicating D217 in a E166-like auxiliary role in catalysis under specific conditions.

**Figure 6:**
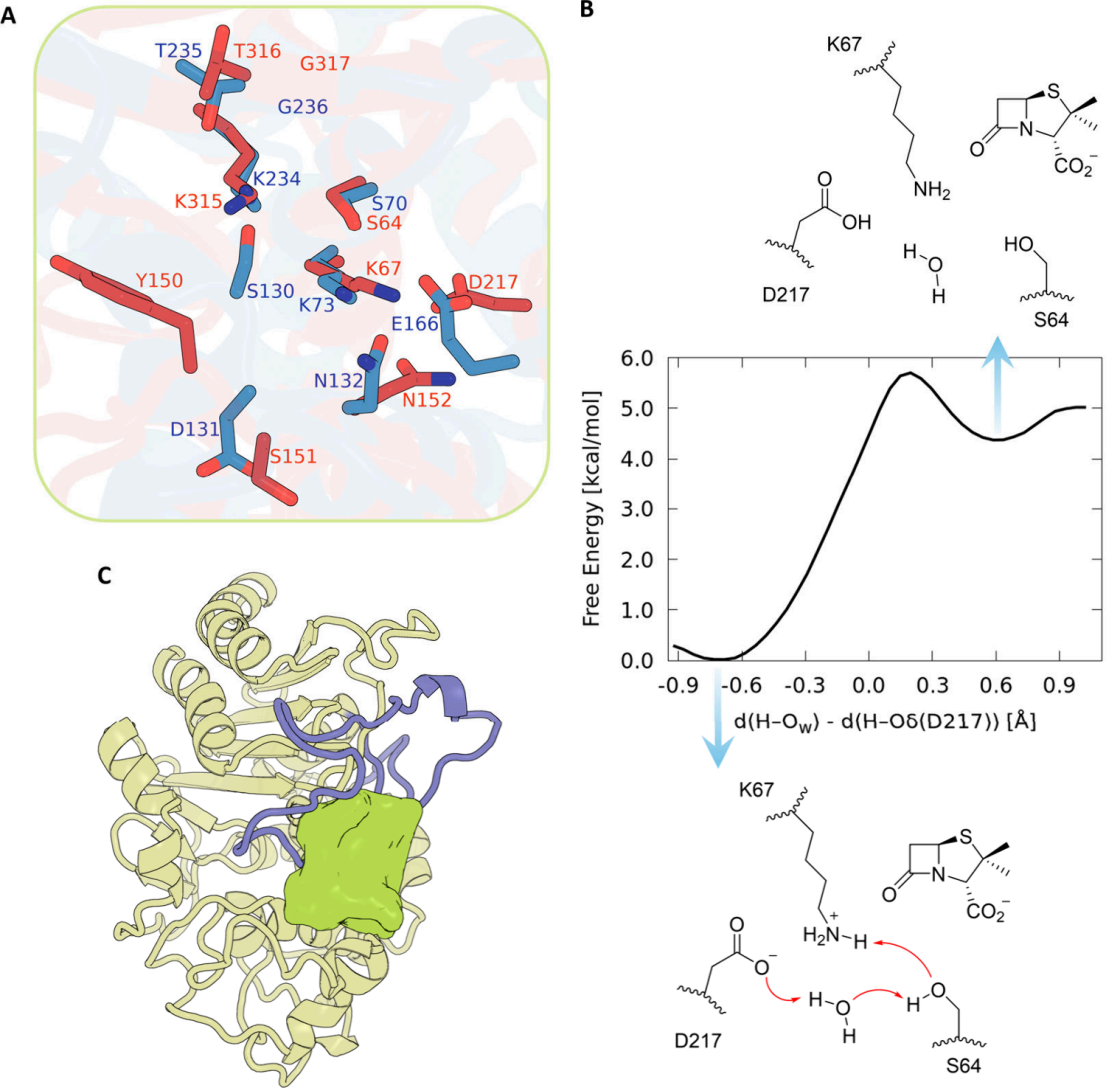
Ω-Loop-mediated functions of PDC-3. (A) Structural alignment of a representative expansive-state conformation of PDC-3 (cluster 4; red) onto the class A β-lactamase KPC-2 crystal structure (PDB entry 5MGI; blue). Conserved catalytic motifs and the side chains of PDC-3 D217 and KPC-2 E166 are shown as sticks, illustrating their analogous positions and potential functional mimicry. (B) Free energy profile for the water-mediated proton transfer from K67 to D217 in PDC-3. (C) Representative constricted-state conformation of PDC-3 (cluster 9; chartreuse). The Ω-loop (purple) shifts inward to occlude the catalytic site, revealing a previously unobserved cryptic pocket on the Ω-loop’s exterior, depicted as a lime-colored surface.

To further probe this possibility, we performed QM/MM simulations starting from the noncovalent Michaelis complex of PDC-3 with penicillanic acid in a representative expansive Ω-loop conformation. In this state, the side chains of K67 and D217 are brought into close proximity, with a K67 Nζ–D217 Oδ distance of ∼2.9 Å, suggesting that a direct proton transfer from K67 to D217 might be structurally plausible. However, our QM/MM free-energy scan along a reaction coordinate defined as the difference between the H–Nζ­(K67) and H–Oδ­(D217) distances shows that, as the proton is moved from K67 to D217, the energy rises by ∼5 kcal/mol without revealing a stable minimum for the D217-protonated species (Figure S29). This behavior parallels earlier QM/MM work on class A β-lactamase TEM-1, where a direct K73→E166 hop was also found to be energetically disfavored and lacked a stable minimum for the protonated glutamate.^27^ Guided by these class A results, where the K73→E166 proton transfer instead proceeds via the catalytic serine and a bridging water molecule to generate a protonated E166, neutral S70, and deprotonated K73 ensemble, we next examined an analogous water-mediated relay in PDC-3. To this end, we defined a reaction coordinate based on the difference between the distances of the transferring proton to the bridging water oxygen and to the D217 Oδ (Figure 6B). Although this collective variable constrains only the H_2_O→D217 step, inspection of the QM/MM trajectories reveals spontaneous proton migration from K67 to S64 and from S64 to the bridging water, exactly mirroring the relay observed in class A β-lactamases.^27,28^ The resulting free energy reveals a low-barrier (≈5–6 kcal/mol) relay K67 → S64 → H_2_O → D217, with a shallow product-like minimum in which the proton resides on D217. Thus, in expansive Ω-loop conformations, D217 can be transiently protonated via a K67→S64→H_2_O→D217 relay and assumes an E166-like general-base role.

In clusters 7–10, specific backbone torsion changes in variants at residues 219 and 221 (E219A, E219 K, and Y221A) can lead to a collapse of the catalytic pocket, wherein the two loops draw closer and constrict the active-site cleft. For instance, the E219 K substitution (cluster 7) prompts the Ω-loop to move dramatically toward helix 11 (Figure 5A and E). This shift disrupts the native contacts involving residues P215 and L216; instead, P215 forms hydrophobic contacts with residues L119 of the P2-loop and L293 on Helix 10, while D217 establishes hydrogen bonds with residues S64, S318, and T319. Cluster 8 exhibits a similar ‘hydrogen-bond and hydrophobic lock as cluster 7 but the Ω-loop moves closer to Helix 10 (Figure 5B and F). Variant E219A adopts another distinct constricted conformation (cluster 9), further shifting the loop toward helix 10. Here, D217 forms a salt bridge with R349 (Figure 5C and G). Concurrently, P215 and L216 form a robust hydrophobic network with L119 on the P2-loop, as well as with A292 and L293 on the R2 loop and Y150 on the conserved catalytic site. In the Y221A variant (cluster 10), backbone torsion changes of residue 221 enable residue E219 to form hydrogen bonds with S64 and S318 (Figure 5D and H).

The collapsed conformation severely restricts access to the catalytic serine, effectively excluding substrates from the active site. Intriguingly, the catalytic-site occlusion simultaneously reveals a previously unobserved cryptic pocket on the opposite side of the Ω-loop (Figure 6C). Conformations of E219 K variants that display this pocket occupy more than half of the ensemble (Table S4), indicating that the cryptic pocket corresponds to a recurrent metastable state rather than a rare isolated fluctuation. This cryptic site presents an appealing target for allosteric inhibition. Designing inhibitors to specifically target this external pocket could stabilize the enzyme in a catalytically inactive conformation, preventing effective binding and hydrolysis of β-lactam antibiotics. Cryptic pockets induced by displacement of the Ω-loop have previously been reported for class A β-lactamases TEM-1 and CTX-M-9.^4,29^ Additionally, alternative conformations involving substantial rearrangements of the Ω-loop have been observed in the class C *Enterobacter cloacae* GC1 enzyme, resulting in cryptic pocket formation that enlarges the substrate-binding region.^30^ However, prior reports predominantly characterized these cryptic pockets as subsites expanding the existing active site. To our knowledge, our findings represent the first documented case where displacement of the Ω-loop completely occludes the catalytic pocket (Figure 6C), thus unveiling an entirely novel cryptic binding site. Although this new cryptic binding site is detected in only three variants, β-lactamases constitute a very large and diverse enzyme family. The Beta-Lactamase DataBase (http://bldb.eu/) currently catalogues 12,422 distinct β-lactamases, of which 6,796 belong to class C and share a conserved fold and catalytic architecture.^31^ Given this high degree of structural conservation, it is plausible that additional class C members may access analogous cryptic openings.

## Conclusions

This study presents an integrated workflow combining well-tempered metadynamics with an unsupervised deep clustering pipeline (CVAE→UMAP→HDBSCAN), successfully capturing the full conformational landscape of the Ω-loop in PDC-3. Compared to conventional long-time scale unbiased simulations, this strategy efficiently crosses high free-energy barriers at an affordable computational cost and automatically categorizes the extensive trajectory data into clearly distinguishable subensembles, laying a solid foundation for subsequent structural analysis. Importantly, the deep clustering pipeline that we propose is not specific to the particular β-lactamase system studied here. In this work, the only system-dependent component is the choice of residues used to construct the distance matrices. Although the specific trained weights obtained here are not intended to generalize to proteins with substantially different topologies or residue mappings, the conceptual approach is transferable across systems. For any protein in which functionally relevant motifs or flexible elements can be identified, an analogous structural descriptor can be constructed and supplied with the same CVAE architecture. In this sense, the CVAE–UMAP–HDBSCAN pipeline provides a general strategy for learning low-dimensional embeddings of conformational ensembles and for automatically identifying metastable states across diverse protein systems.

Dynamic analyses reveal that backbone torsions of residues 219 and 221, whether wild-type or variants, act as key switches that drive large-scale expansion and constriction of the active site. By rewiring the surrounding hydrogen-bond and hydrophobic networks, these residues finely control substrate access, directly influencing enzymatic activity. In contrast, residues 211 and 214 primarily influence local flexibility without reshaping the overall topology. Notably, residue D217 consistently establishes a stable salt bridge with K67 across numerous expansive conformations, occupying a spatial position highly analogous to that of the general base residue E166 in class A β-lactamases. This structural analogy raises the intriguing possibility that D217 might transiently serve as an alternative catalytic base under certain conformations and specific substrates. Such an auxiliary catalytic function could explain the observed variations in catalytic efficiency and substrate specificity among class C β-lactamases but also offer valuable guidance for antibiotic selection and personalized treatment strategies, providing new insights into their evolutionary adaptation and clinical implications. Furthermore, in conformational clusters where inward displacement of the Ω-loop leads to active-site occlusion, we identified a previously unobserved cavity located on the outer side of the Ω-loop. Although physically distant from the catalytic serine, this cavity might be a novel target for noncompetitive inhibitors, capable of locking the enzyme in an inactive conformation and blocking antibiotic hydrolysis. However, there is a substantial gap between identifying a cryptic pocket *in silico* and establishing it as a practical drug target. the present work provides a structural and mechanistic rationale for future work.

Moreover, cryptic binding sites offer valuable opportunities for addressing antibiotic resistance. Such cryptic pockets provide new intervention points that traditional structure-based designs focused on only static active sites would miss. This strategy broadens the chemical space for inhibitor design and enables conformation-selective inhibition, which may help retain potency against resistant variants.^32^ To identify such cryptic pockets, specialized methods are required because these hidden states may not be detected under traditional experimental conditions.^33^ Molecular dynamics (MD) simulations such as unbiased long-time scale MD, enhanced sampling techniques, and mixed-solvent simulations have each successfully uncovered cryptic binding pockets and characterized their opening mechanisms.^33^ More recently, machine-learning approaches have emerged to predict ligand-induced conformational changes directly from static structures.^34^ Incorporating these modern computational tools alongside traditional MD will further enhance our ability to detect cryptic sites and exploit them in the fight against antibiotic resistance.

Overall, this research not only sheds new light on the dynamic mechanisms underlying antibiotic resistance in PDC-3 but also demonstrates the broad applicability and potential of the integrated ‘enhanced sampling + deep clustering’ methodology. Furthermore, it provides valuable methodological insights that can be transferred to the exploration of cryptic conformational states in other highly dynamic proteins, including kinase activation loops and GPCR extracellular regions.

## Methods and Materials

### Well-Tempered Metadynamics Simulations

The structure of the PDC-3 β-lactamase was prepared starting from the Protein Data Bank (PDB ID: 8SDR).^35^ Nine variants (V211A, V211G, G214A, G214R, E219A, E219G, E219 K, Y221A, and Y221H), detected in highly drug-resistant *P. aeruginosa* clinical isolates, were constructed *in silico* using the ICM mutagenesis program.^36^ All systems were parametrized according to the high-throughput molecular dynamics (HTMD) protocol.^37^ Protonation states were assigned with proteinprepare in the Moleculekit module of HTMD. The Amber ff14SB force field described the protein, combined with an explicit TIP3P water model.^38,39^ Each system was energy-minimized with 3,000 steps of the steepest-descent integrator and then equilibrated in the NPT ensemble for 5 ns. Temperature (300 K) and pressure (1 bar) were maintained by a Langevin thermostat and a Berendsen barostat, respectively.^40^ A 4 fs integration step was used. Well-tempered metadynamics (WT-MetaD) was used to enhance sampling starting from the equilibrated structures of wild-type PDC-3 and each variant. Simulations were run using ACEMD program combined with the PLUMED 1.3 plugin.^41–43^

To probe how mutations influence protein dynamics, we chose the backbone ϕ and ψ dihedral angles of selected residues as collective variables (CVs). For wild-type PDC-3, four independent WT-MetaD simulations were performed, each using the ϕ and ψ angles of one residue (V211, G214, E219, or Y221) as CVs; for each variant, the corresponding mutated residue’s backbone dihedrals served as CVs. Gaussian hills were deposited on these two CVs with a width of 0.1 rad and a height of 0.5 kJ/mol. Gaussians were added every 4 ps, giving a deposition rate of 0.125 kJ/(mol·ps). The bias factor was set to 15. After 3 μs of WT-MetaD in the NVT ensemble, the free energy surface converged. This was determined based on (a) the diffusive behavior along CV1 ϕ and CV2 ψ, (b) converged FES plots for CV1 ϕ and CV2 ψ, and (c) the deposition of the Gaussian bias potentials (Figures S1–S13). The simulation length represents the sampling time when all three parameters were converged. The two-dimensional free energy landscape along the chosen CVs was reconstructed by integrating the deposited bias along the trajectory. Errors on minima and barriers were estimated from the largest variation observed in the monodimensional projections over the final 100 ns of simulation, amounting to ± 0.5 kJ/mol. For each system, we shifted the reconstructed free-energy profile so that its maximum value is set to 0 kJ/mol. All structures from trajectories with free energy below – 200 kJ/mol were extracted for further analysis.

### Convolutional Variational Autoencoder Model Construction

From each WT-MetaD trajectory, we extracted only those frames whose free energy fell below −200 kJ/mol, ensuring that our analysis focused on stable, functionally relevant conformations. From each low-energy snapshot, we built a 53 × 53 distance matrix by measuring pairwise Cα distances among residues in (1) the three conserved catalytic motifs: S64–K67, Y150–N152, and K315-G317; (2) the Ω-loop segment L209-S226; (3) helix 10 residues L280-Q294; and (4) additional residues lining or near the active site (L119, Q120, N314, S318, T319, R342, N343, P345, N346, and R349). A full list of 53 residues is provided in Supplementary Note 3. The 53 × 53 matrices were padded with one row and one column of zeros to yield 54 × 54 arrays, which are convenient for the convolutional architecture. Before training, all distances were rescaled to the [0, 1] interval by dividing by the maximum distance observed in the entire data set. Each distance matrix thus provides a 2D “image” of the protein conformation in which local contact patterns report on Ω-loop positioning and broader tertiary rearrangements.

To learn a compact representation of these conformations, we trained a CVAE on the collection of distance matrices generated from all variants using TensorFlow 2.4.0.^17,44^ The CVAE’s encoder consists of three convolutional layers (with feature maps of 32, 64, and 128 and filter sizes 3 × 3, strides of (1,1), (1,1), and (2,2) respectively) followed by one dense layer of 32 neurons with 0.3 dropout; the decoder mirrors this architecture in reverse. We configured the model with a single input channel, a batch size of 512, and an 8-dimensional latent space. The model was trained for 100 epochs by using the RMSprop optimizer. During training, 20% of the data was used only for validation. The loss function was defined as

$$L_{\mathrm{VAE}}\left(x,\mathrm{x̂}\right)=\alpha \cdot \mathrm{MSE}\left(x,\mathrm{x̂}\right)+\mathrm{KL}\left(q\left(z|x\right)∥p\left(z\right)\right)$$

where the first term is the mean squared error between each flattened input distance matrix *x* and its reconstruction *x̂*, and the second term is the Kullback–Leibler divergence enforcing a Gaussian prior *p*(*z*) on the latent variables *z*. The α = 200 balances reconstruction fidelity against the RMSD values.

Once trained, the CVAE encoder maps each distance matrix onto an 8-dimensional latent vector that captures the principal conformational variations of the Ω-loop and surrounding structural motifs. To visualize and cluster these embeddings, we applied UMAP to reduce the 8-dimensional latent vectors to two dimensions.^18^ Finally, we employed HDBSCAN on the 2D UMAP embeddings to identify distinct conformational states of the Ω-loop.^19,20^ Because HDBSCAN can handle irregular cluster shapes, it effectively separates the conformational ensemble into discrete metastable basins for further analysis.

### Structural Analysis

All RMSD and RMSF calculations were performed on frames whose free energy was less than −200 kJ/mol. Using MDTraj, each selected frame was aligned to the equilibrated reference structure through a least-squares fitting procedure applied to all Cα atoms.^45^ RMSD was then computed as the root-mean-square deviation of those aligned Cα positions from their reference coordinates.^46^ For the RMSF analysis, we measured the root-mean-square fluctuation of each residue’s Cα position relative to its reference coordinate across all aligned, low-energy frames.^46^ Additionally, side chain χ_1_ torsion angles were extracted for all residues except alanine and glycine from each frame. To quantify how mutations alter the distribution of side chain torsion angles, we calculated the Jensen-Shannon (JS) divergence between the χ_1_ angle distributions of wild-type PDC-3 and each variant.^47^ For two discrete probability distributions *P* and *Q* defined over the same support Ω, the Kullback–Leibler divergence is

$$\mathrm{KL}\left(P∥Q\right)=\underset{x\in \Omega }{\sum }P\left(x\right)\log\left(\frac{P\left(x\right)}{Q\left(x\right)}\right)$$

and the Jensen-Shannon divergence is

$$\mathrm{JS}\left(P∥Q\right)=\frac{1}{2}\mathrm{KL}\left(P∥\frac{P+Q}{2}\right)+\frac{1}{2}\mathrm{KL}\left(Q∥\frac{P+Q}{2}\right)$$

The interactions were quantified using MDAnalysis and custom scripts.^48^ Hydrogen bonds were identified when a donor–acceptor pair (nitrogen or oxygen atoms) satisfied a distance cutoff of 3.5 Å and a donor-hydrogen-acceptor angle above 150^◦^. Salt bridges were defined for any Asp/Glu oxygen atom and Lys/Arg nitrogen atom separated by less than 4.0 Å. Hydrophobic contacts were counted between heavy atoms of nonpolar residues (Ala, Val, Leu, Ile, Phe, Met, Pro, Trp, and Tyr) whenever their interatomic distance fell below 4.0 Å.

### QM/MM Setup and Umbrella Sampling

Representative conformation of PDC-3 in which K67 and D217 formed a salt bridge was selected from the enhanced-sampling simulations, and penicillanic acid was then placed into the active site to construct noncovalent Michaelis complexes of PDC-3 with the β-lactam substrate, which provided the Cartesian coordinates for the subsequent QM/MM calculations. The protein was described with the AMBER ff14SB force field^49^ and water with the TIP3P model,^50^ while penicillanic acid was parametrized with GAFF2.^51^ The complex was solvated in a rectangular TIP3P water box with a ∼ 10 Å buffer and neutralized with Na^+^/Cl^–^ counterions. The solvated system was minimized, then heated from 0 to 300 K over 500 ps and equilibrated for 2 ns at 300 K. All MD simulations were carried out with AMBER 22.^38^ The final equilibrated snapshot provided the initial coordinates for the QM/MM simulations.

QM/MM simulations were performed with Amber22 at 300 K in a constant volume ensemble with a 1 fs time step. All water molecules within 20 Å of the penicillanate substrate were kept for the QM/MM calculations and were included in the MM region (with the exception of the catalytic water molecule, which was included in the QM region). The QM region comprised the entire penicillanic acid molecule, the side chains of S64, K67 and D217, and the bridging water that mediates proton transfer. The QM subsystem was treated at the PM6 semiempirical level,^52^ and an electrostatic embedding scheme was used to couple the QM and MM regions.^53^ Two one-dimensional reaction coordinates were considered. For the direct K67 to D217 pathway, the coordinate was defined as the distance between the transferring proton and the Nζ atom of K67 minus the distance between the same proton and the Oδ atom of D217. For the water-mediated relay, the coordinate was defined as the distance between the transferring proton and oxygen of the catalytic bridging water minus the distance between the same proton and the Oδ atom of D217. For each reaction coordinate, umbrella windows were placed every 0.30 Å. Each window was propagated for 5 ps of QM/MM molecular dynamics. The potential of the mean force at 300 K was then reconstructed using the 1d WHAM implementation of Grossfield.

### Protein Expression and Crude Extract Cell Lysis

PDC-3 and variants were expressed in *E. coli* DH10B background and pBC SK (−) vector by Celtek Bioscience as previously described in our previous work.^9,12^ Cells were grown in 500 mL of super optimal broth (SOB) at 37 °C overnight. The cell pellets were generated by centrifugation and frozen at 20 °C for 12 h prior to a 40 min lysis in 50 mM Tris-HCl buffer (pH 7.4) containing 40 mg/mL lysozyme, 0.1 mM magnesium sulfate, 250 U benzonase nuclease, and 1 mM ethylenediaminetetraacetic acid (EDTA). The supernatant was filtrated and buffer exchanged in PBS, pH7.4.

### Steady-State Kinetics

Steady-state kinetic and inhibitor parameters were determined by using an Agilent 8453 diode array spectrophotometer at room temperature as previously described.^9,12,21,54^ Each assay was performed in 10 mM phosphate-buffered saline (PBS) at pH 7.4 at room temperature (RT 25 °C) in a quartz cuvette with a 1 cm path length. Nitrocefin (NCF), a readily hydrolyzed chromogenic cephalosporin, was used as the reporter substrate. The kinetic parameters for NCF (*V*_max_ and *K*_m_) were obtained by nonlinear least-squares fitting of the data to the Henri-Michaelis–Menten equation

$$v=\frac{V_{\max}\left[\mathrm{NCF}\right]}{K_{m}+\left[\mathrm{NCF}\right]}$$

using Origin 7.5VR (OriginLab, Northampton, MA).

For ceftazidime (CAZ) and ceftolozane (TOL), we employed a competition assay in which CAZ or TOL was treated as competitive inhibitor of nitrocefin (NCF) hydrolysis, yielding a *K*_i, app_ that is expected to closely approximate the *K*_m_.^55,56^ As previously reported in our papers,^9,12^ NCF was used at a concentration equal to 5 × *K*_m,NCF_ and initial velocities were measured during the first 60 s in the presence of increasing concentrations of CAZ or TOL. Initial velocities (recorded during the first 10–60 s) were plotted as a function of inhibitor concentration, and the apparent inhibition parameter *K*_i, app, obs_ was obtained from linear fits as the ratio of the slope to the *y* intercept. For all linear regressions, the R^2^ values were more than 0.95.

To account for the occupancy and affinity of NCF at the active site under the assay conditions, the observed apparent inhibition constant was corrected according to

$$K_{i,\mathrm{app}}=\frac{K_{i,\mathrm{app},\mathrm{obs}}}{1+\left(\frac{\left[\mathrm{NCF}\right]}{K_{m,\mathrm{NCF}}}\right)}$$

The resulting corrected values (*K*_i,CAZ_ and *K*_i,TOL_) represent the apparent affinity of ceftazidime or ceftolozane for the NCF-occupied active site. We followed the same experimental conditions as previously described in our studies,^9,12^ using equivalent volume of periplasmic lysates normalized to yield similar velocity (*V*_max_ ∼ 1 initial rate/unit based on the initial rates of NCF hydrolysis) consistent with the conditions used for *K*_m,NCF_ determination for each variant.

## Supplementary Material







## Data Availability

The simulation input files, including the AMBER topology (.prmtop) and coordinate (.inpcrd) files, as well as the representative cluster structures (.pdb), have been deposited in Zenodo (10.5281/zenodo.15948375).

## Notes

Supplementary Notes 1–3 details occupanices of residue-residue interactions, details from Beta-Lactamase DataBase and a full list of residues used to create the distance matrices; Figures S1–S29 illustrate convergence metrics from WT-MetaD simualtions, Free Energy Landscapes, RMSD and RMSF plots, Side chain torsion angle distributions, , Jensen-Shannon divergence, Inhibition constants for ceftazidime and ceftolozane, CVAE training and validation loss convergence, Simulation frame populations, Hydrogen bond distances, Free energy profiles and Tables S1–S5 tabulate Frame counts per system, Jensen-Shannon divergence, Population of simulation frames, Quantitative characterisation of conformational differences (PDF)
Supplementary Data 1 is residue-residue interaction occupancies from enhanced sampling (CSV)
Supplementary Data 2 is the multiple sequence alignment of class C β-lactamases from BLDB (CSV)
