# Supplementary material for: Cryptic Binding Pockets in PDC‑3 β‑Lactamase Modulate Resistance Profiles
Source: JACS Au. 2026 Feb 20;6(3):1833–46. doi: 10.1021/jacsau.5c01707 (PMC13014224; doi:10.1021/jacsau.5c01707)
Supplement: Supplementary file 1 [file au5c01707_si_001.pdf]

Supporting Information

Cryptic Binding Pockets in PDC-3 β-Lactamase Modulate Resistance Profiles

Shuang Chen[1], Fedaa Attana[1,7], Andrea M. Hujer,[2] Christopher R Bethel,[2] Magdalena A. Taracila,[2] Robert A. Bonomo[2,3,4,5,6], and Shozeb Haider [1,7,*]

[1] UCL School of Pharmacy, London, WC1N 1AX, UK

[2] Research Service, Louis Stokes Cleveland Department of Veterans Affairs Medical Center, Cleveland, OH 44106-1702, USA

[3] Department of Molecular Biology and Microbiology, Case Western Reserve University School of Medicine, Cleveland, OH 44106-5029, USA

[4] Department of Medicine, Case Western Reserve University School of Medicine, Cleveland, OH 44106-5029, USA

[5] Departments of Pharmacology, Biochemistry, and Proteomics and Bioinformatics, Case Western Reserve University School of Medicine, Cleveland, OH 44106-5029, USA

[6] CWRU-Cleveland VAMC Center for Antimicrobial Resistance and Epidemiology (Case VA CARES), Cleveland, OH 44106-5029, USA

[7] Prince Fahd Bin Sultan Chair for Biomedical Research (PFSCBR), University of Tabuk, Tabuk 71491, Saudi Arabia

- Corresponding Author
  Prof Shozeb Haider (shozeb.haider@ucl.ac.uk)
  ORCID: 0000-0003-2650-2925

Supplementary Note 1

To identify function-related conformational changes, we quantified the occupancies of residue–residue interactions for each cluster using the full enhanced-sampling dataset (13 trajectories × 30,000 frames; 390,000 frames total; see Supplementary Data 1). For every frame, we detected all hydrogen bonds (hb), salt bridges (sb), hydrophobic contacts (hc), cation–π interactions (cp), and π–π stacking interactions (pp) across the protein, and then, for each cluster and each system, computed the fraction of frames in which a given interaction is present.

Supplementary Note 2

Class C β-lactamase sequences were obtained from the Beta-Lactamase DataBase (BLDB; http://bldb.eu/). On 31 July 2025, BLDB listed 6,785 entries annotated as class C β-lactamases, of which 6,688 entries had an associated amino-acid sequence available for download. These 6,688 class C sequences were exported in FASTA format and used for the multiple sequence alignment (MSA) reported in this work. The resulting multiple sequence alignment is provided as Supplementary Data 2.

Supplementary Note 3

The full list of the 53 residues used to construct the distance matrices is: S64, V65, S66, K67, L119, Q120, Y150, S151, N152, L209, R210, V211, G212, P213, G214, P215, L216, D217, A218, E219, G220, Y221, G222, V223, K224, T225, S226, L280, K281, R282, L283, Q284, A285, G286, N287, S288, T289, P290, M291, A292, L293, Q294, N314, K315, T316, G317, S318, T319, R342, N343, P345, N346, and R349

Supplementary Data 1 System- and cluster-resolved residue–residue interaction occupancies from enhanced sampling.

Supplementary Data 2 Multiple sequence alignment of class C β-lactamases from BLDB.

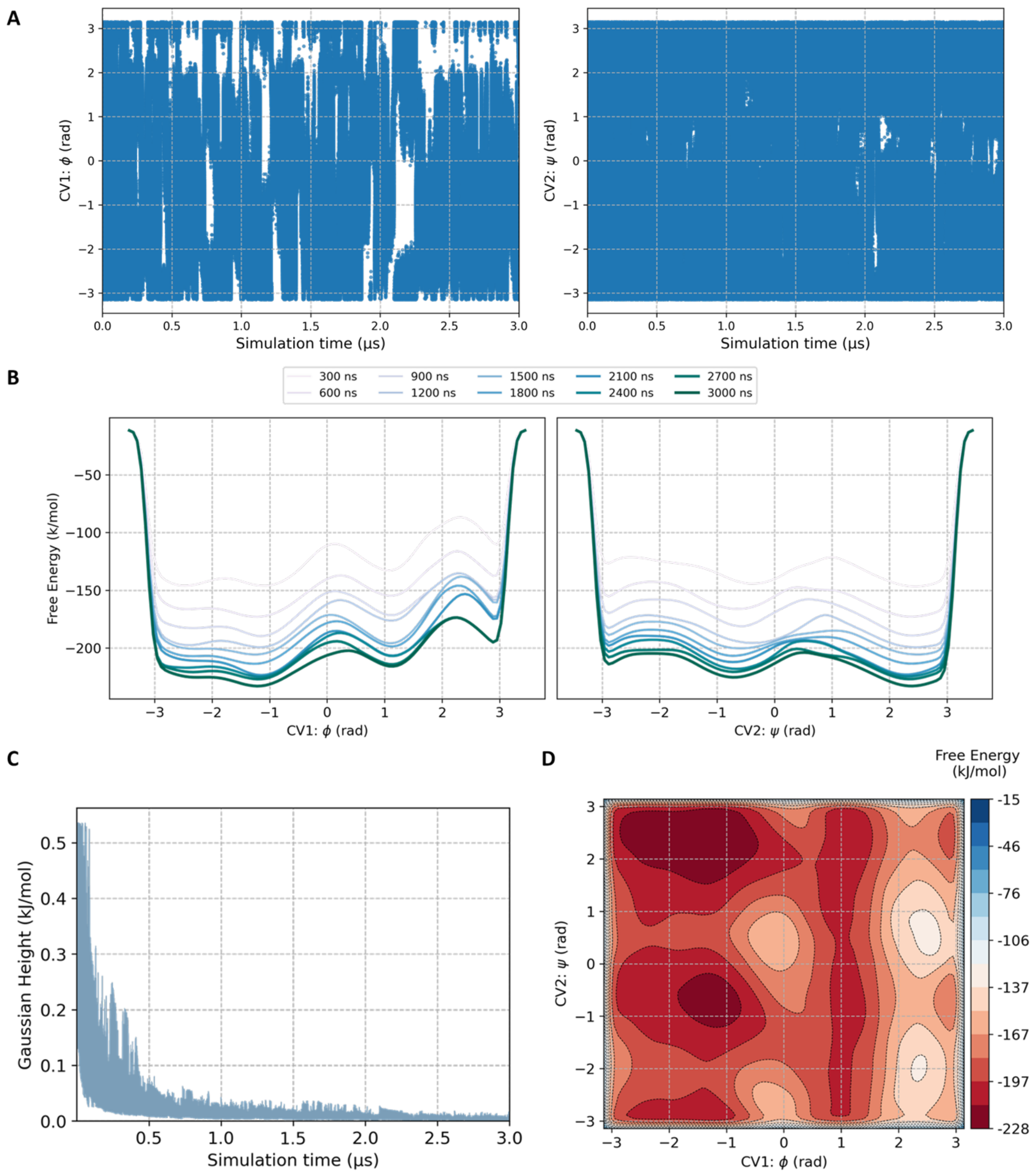

Figure S1. Metadynamics simulation results for wild-type PDC-3 (V211 system). (A) Time evolution of the two collective variables (CVs) over the entire 3 μs metadynamics run, showing CV_1 (ϕ, in radians; left) and CV_2 (ψ, in radians; right) as functions of simulation time. (B) One-dimensional free-energy profiles extracted at cumulative metadynamics times of 300–3000 ns for CV_1 (left) and CV_2 (right). (C) Height of added Gaussian bias potentials as a function of simulation time. The Gaussian height decays rapidly from approximately 0.5 kJ/mol toward near zero by ~3 μs. (D) Final two-dimensional free-energy surface (FES) reconstructed from metadynamics, plotted with CV_1 (ϕ, in radians) on the horizontal axis and CV_2 (ψ, in radians) on the vertical axis. The color scale (right) denotes relative free energy in kJ/mol: low-energy basins appear in dark shades, whereas high-energy regions and barriers appear in lighter shades.

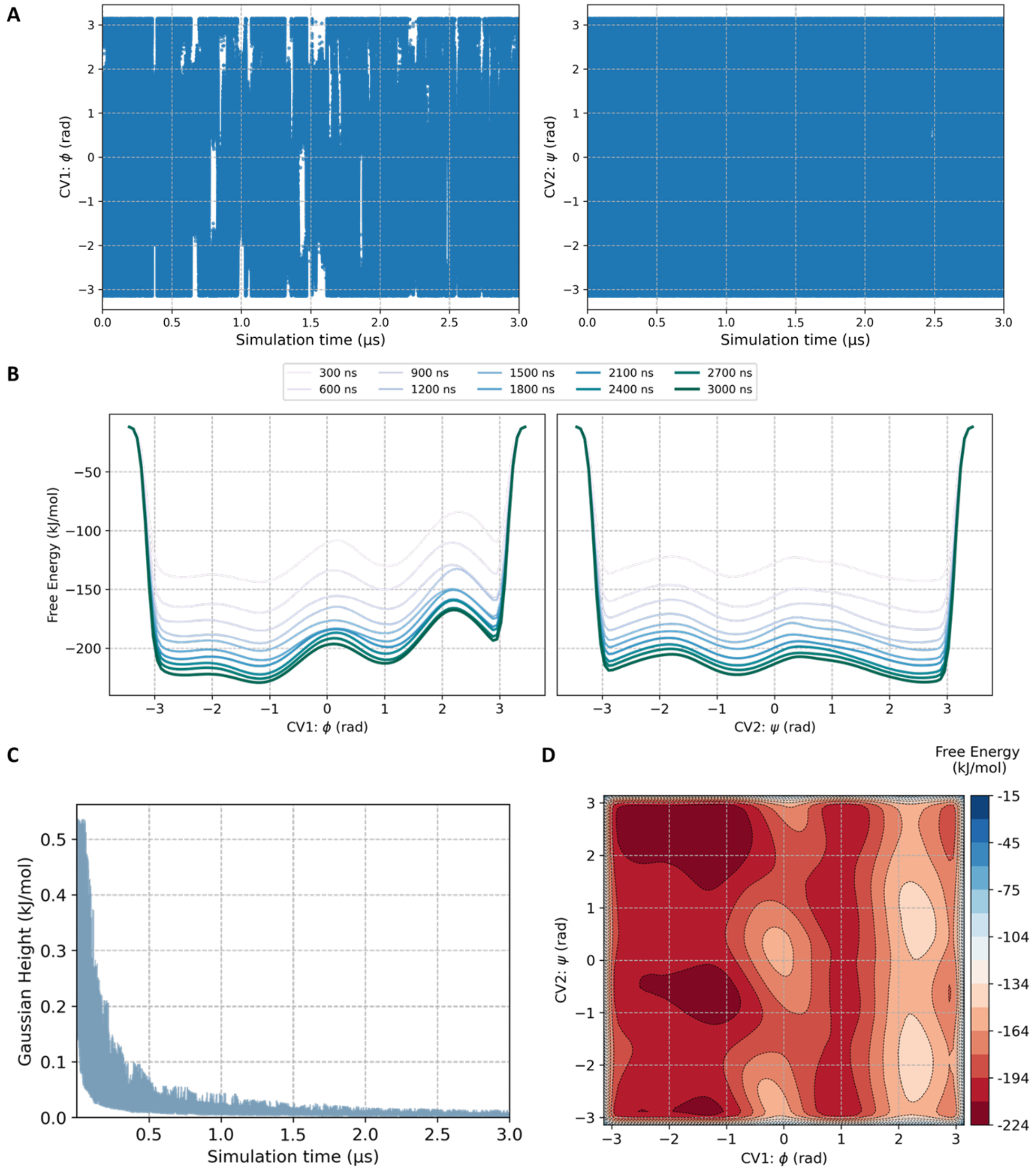

Figure S2. Metadynamics simulation results for V211A variants. (A) Time evolution of the two collective variables (CVs) over the entire 3 μs metadynamics run, showing CV_1 (ϕ, in radians; left) and CV_2 (ψ, in radians; right) as functions of simulation time. (B) One-dimensional free-energy profiles extracted at cumulative metadynamics times of 300–3000 ns for CV_1 (left) and CV_2 (right). (C) Height of added Gaussian bias potentials as a function of simulation time. The Gaussian height decays rapidly from approximately 0.5 kJ/mol toward near zero by $\sim$3 μs. (D) Final two-dimensional free-energy surface (FES) reconstructed from metadynamics, plotted with CV_1 (ϕ, in radians) on the horizontal axis and CV_2 (ψ, in radians) on the vertical axis. The color scale (right) denotes relative free energy in kJ/mol: low-energy basins appear in dark shades, whereas high-energy regions and barriers appear in lighter shades.

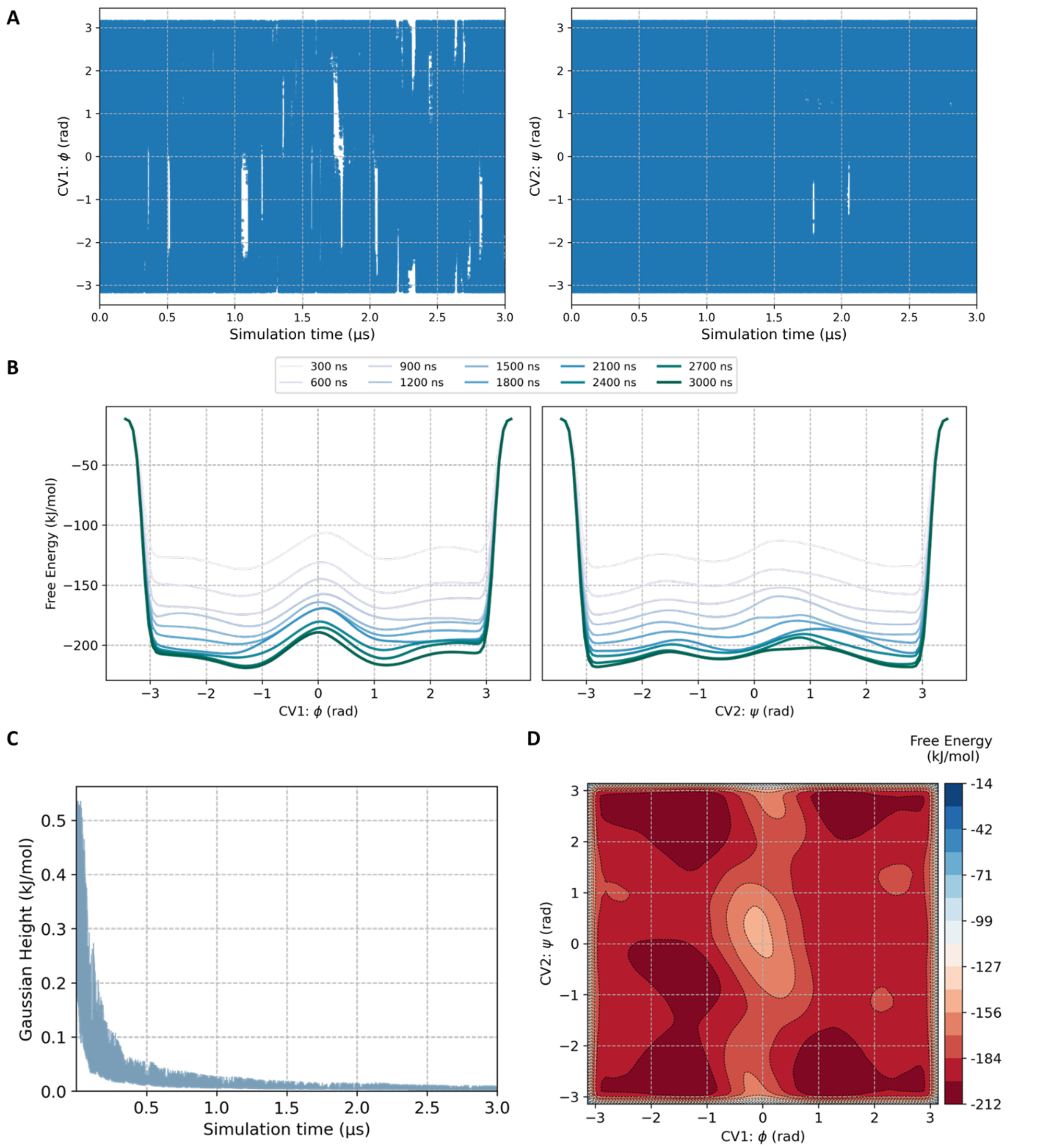

Figure S3. Metadynamics simulation results for V211G variants. (A) Time evolution of the two collective variables (CVs) over the entire 3 μs metadynamics run, showing CV_1 (ϕ, in radians; left) and CV_2 (ψ, in radians; right) as functions of simulation time. (B) One-dimensional free-energy profiles extracted at cumulative metadynamics times of 300–3000 ns for CV_1 (left) and CV_2 (right). (C) Height of added Gaussian bias potentials as a function of simulation time. The Gaussian height decays rapidly from approximately 0.5 kJ/mol toward near zero by ~3 μs. (D) Final two-dimensional free-energy surface (FES) reconstructed from metadynamics, plotted with CV_1 (ϕ, in radians) on the horizontal axis and CV_2 (ψ, in radians) on the vertical axis. The color scale (right) denotes relative free energy in kJ/mol: low-energy basins appear in dark shades, whereas high-energy regions and barriers appear in lighter shades.

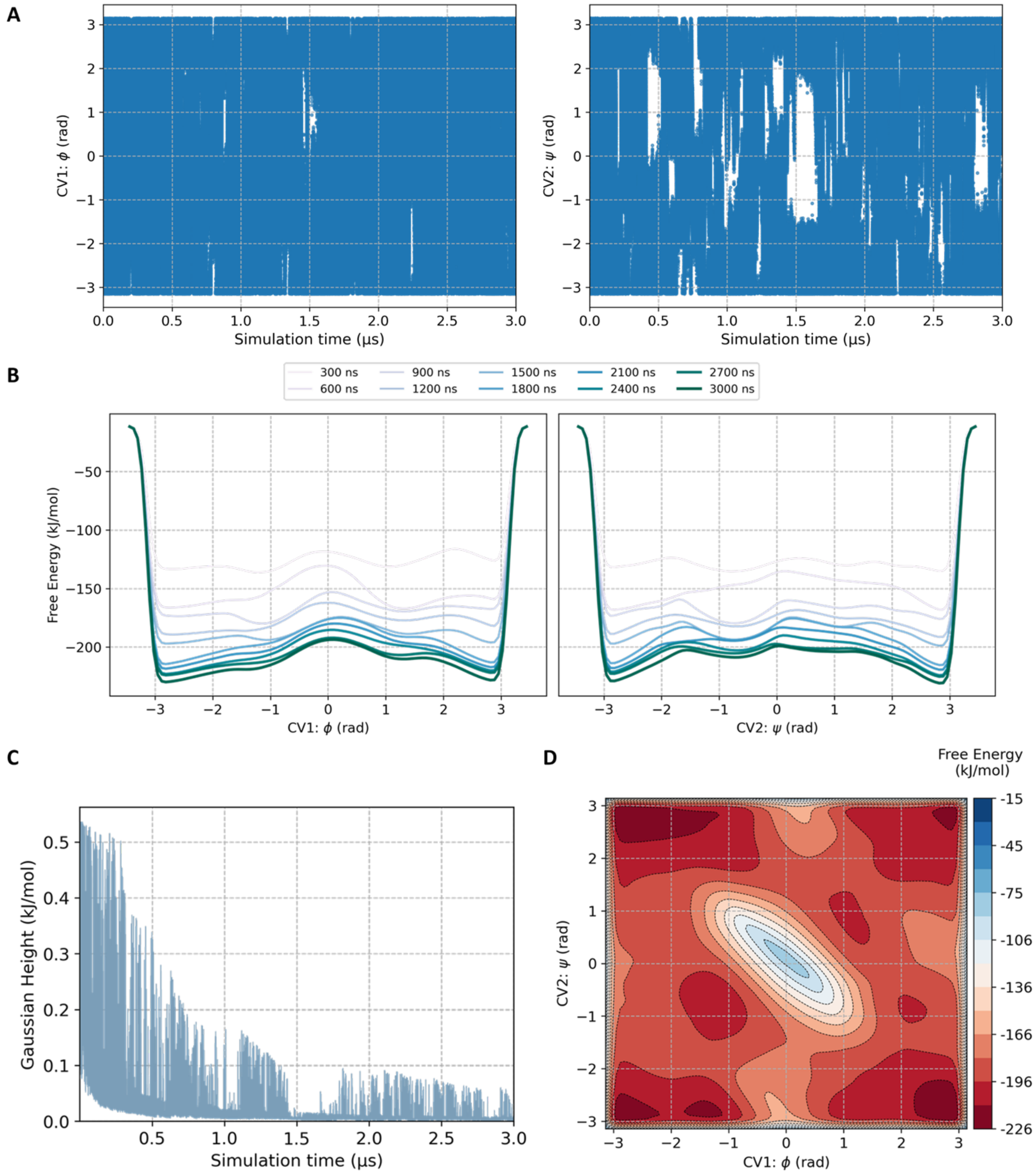

Figure S4. Metadynamics simulation results for wild-type PDC-3 (G214 system). (A) Time evolution of the two collective variables (CVs) over the entire 3 μs metadynamics run, showing CV_1 (ϕ, in radians; left) and CV_2 (ψ, in radians; right) as functions of simulation time. (B) One-dimensional free-energy profiles extracted at cumulative metadynamics times of 300–3000 ns for CV_1 (left) and CV_2 (right). (C) Height of added Gaussian bias potentials as a function of simulation time. The Gaussian height decays rapidly from approximately 0.5 kJ/mol toward near zero by ~3 μs. (D) Final two-dimensional free-energy surface (FES) reconstructed from metadynamics, plotted with CV_1 (ϕ, in radians) on the horizontal axis and CV_2 (ψ, in radians) on the vertical axis. The color scale (right) denotes relative free energy in kJ/mol: low-energy basins appear in dark shades, whereas high-energy regions and barriers appear in lighter shades.

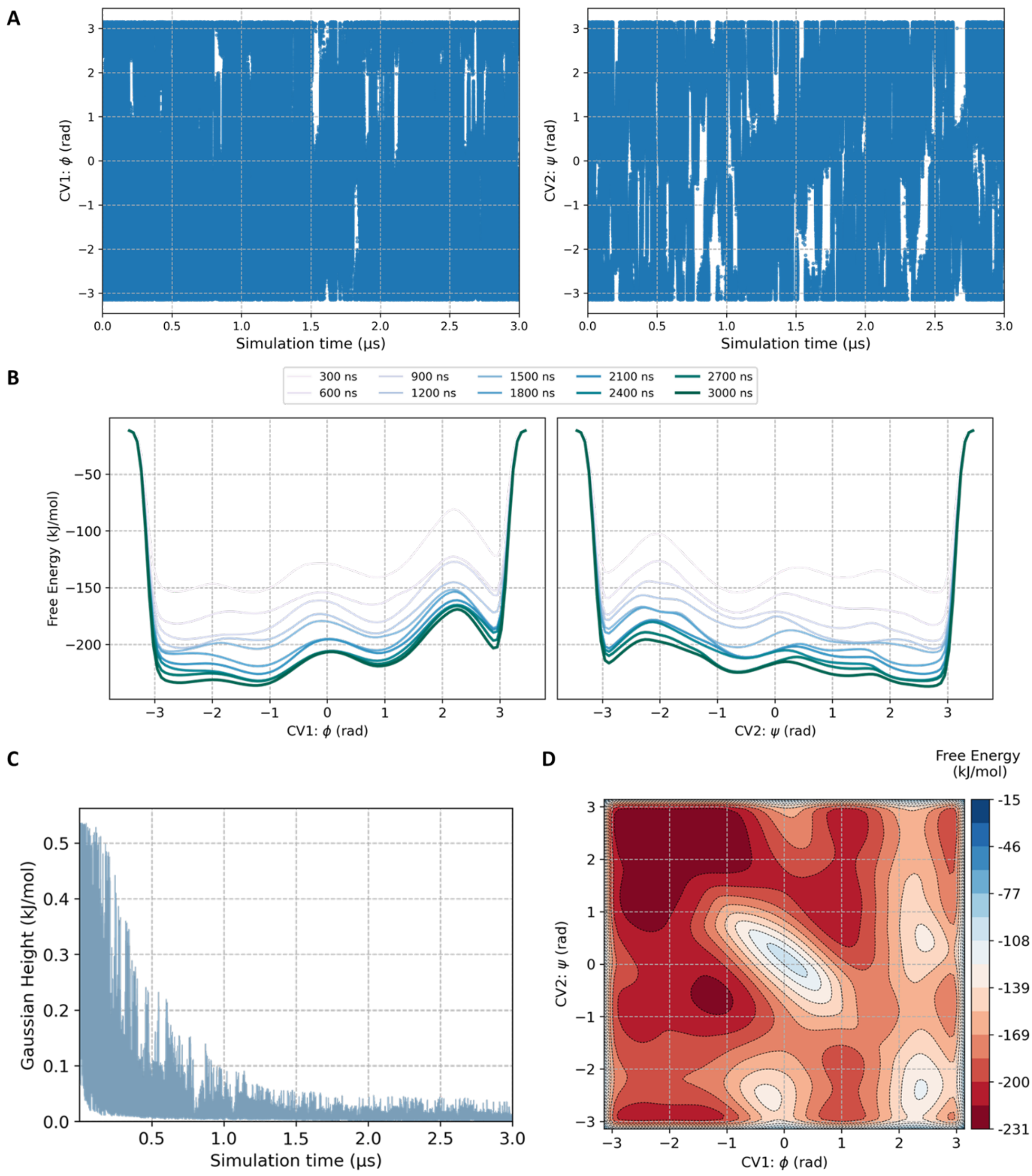

Figure S5. Metadynamics simulation results for G214A variants. (A) Time evolution of the two collective variables (CVs) over the entire 3 μs metadynamics run, showing CV_1 (ϕ, in radians; left) and CV_2 (ψ, in radians; right) as functions of simulation time. (B) One-dimensional free-energy profiles extracted at cumulative metadynamics times of 300–3000 ns for CV_1 (left) and CV_2 (right). (C) Height of added Gaussian bias potentials as a function of simulation time. The Gaussian height decays rapidly from approximately 0.5 kJ/mol toward near zero by $\sim$3 μs. (D) Final two-dimensional free-energy surface (FES) reconstructed from metadynamics, plotted with CV_1 (ϕ, in radians) on the horizontal axis and CV_2 (ψ, in radians) on the vertical axis. The color scale (right) denotes relative free energy in kJ/mol: low-energy basins appear in dark shades, whereas high-energy regions and barriers appear in lighter shades.

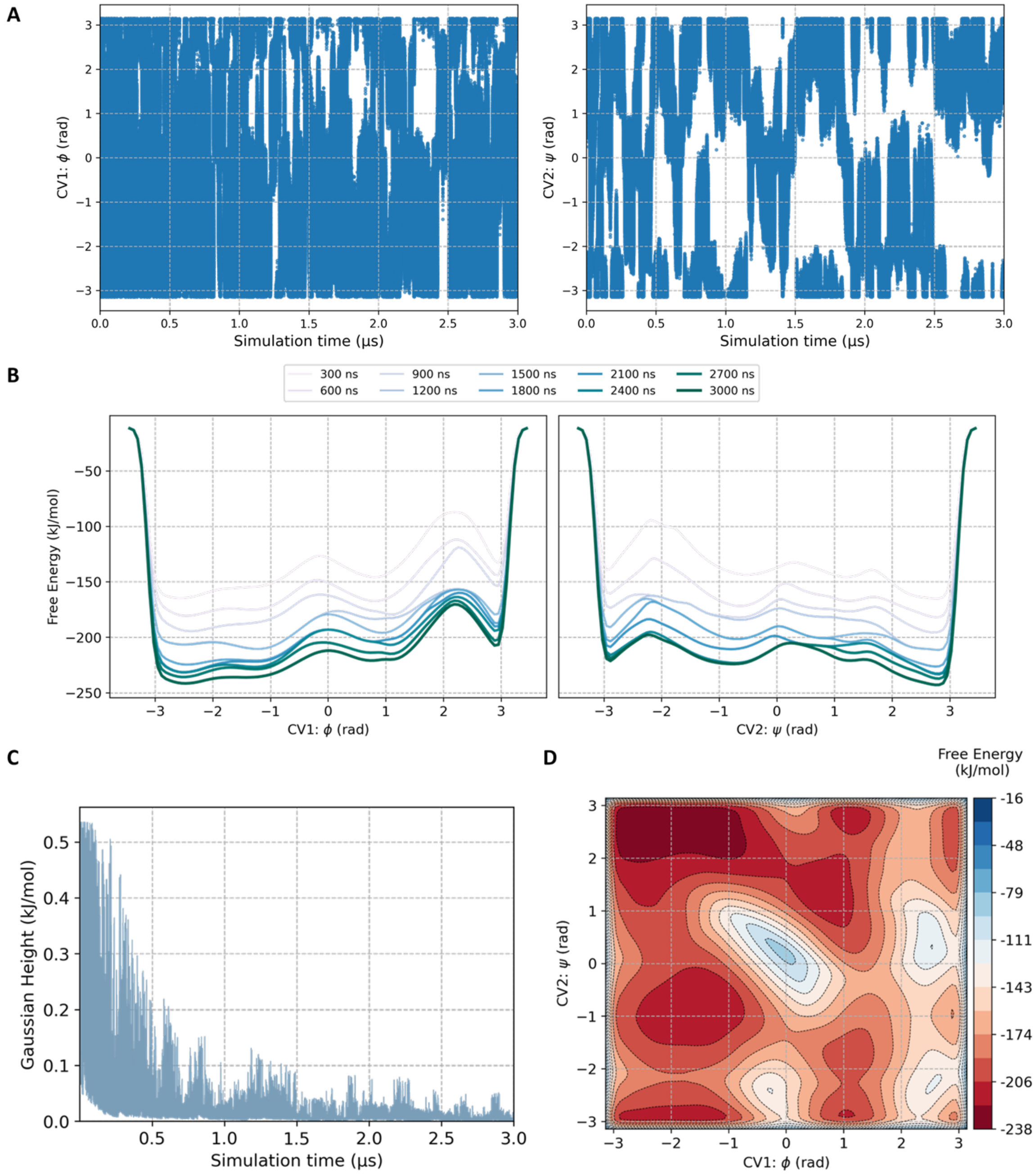

Figure S6. Metadynamics simulation results for G214R variants. (A) Time evolution of the two collective variables (CVs) over the entire 3 μs metadynamics run, showing CV_1 (ϕ, in radians; left) and CV_2 (ψ, in radians; right) as functions of simulation time. (B) One-dimensional free-energy profiles extracted at cumulative metadynamics times of 300–3000 ns for CV_1 (left) and CV_2 (right). (C) Height of added Gaussian bias potentials as a function of simulation time. The Gaussian height decays rapidly from approximately 0.5 kJ/mol toward near zero by ~3 μs. (D) Final two-dimensional free-energy surface (FES) reconstructed from metadynamics, plotted with CV_1 (ϕ, in radians) on the horizontal axis and CV_2 (ψ, in radians) on the vertical axis. The color scale (right) denotes relative free energy in kJ/mol: low-energy basins appear in dark shades, whereas high-energy regions and barriers appear in lighter shades.

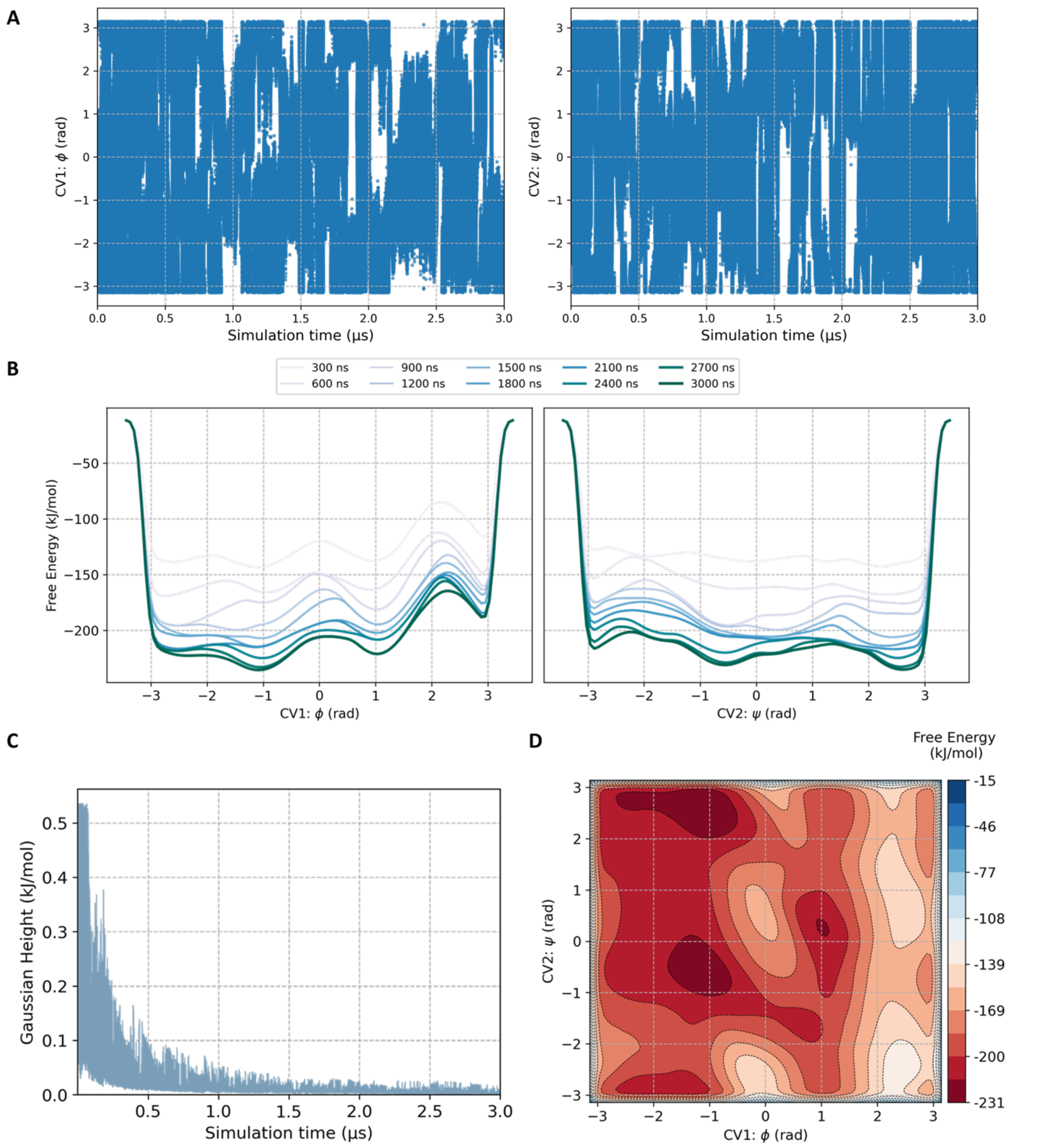

Figure S7. Metadynamics simulation results for wild-type PDC-3 (E219 system). (A) Time evolution of the two collective variables (CVs) over the entire 3 μs metadynamics run, showing CV_1 (ϕ, in radians; left) and CV_2 (ψ, in radians; right) as functions of simulation time. (B) One-dimensional free-energy profiles extracted at cumulative metadynamics times of 300–3000 ns for CV_1 (left) and CV_2 (right). (C) Height of added Gaussian bias potentials as a function of simulation time. The Gaussian height decays rapidly from approximately 0.5 kJ/mol toward near zero by $\sim$3 μs. (D) Final two-dimensional free-energy surface (FES) reconstructed from metadynamics, plotted with CV_1 (ϕ, in radians) on the horizontal axis and CV_2 (ψ, in radians) on the vertical axis. The color scale (right) denotes relative free energy in kJ/mol: low-energy basins appear in dark shades, whereas high-energy regions and barriers appear in lighter shades.

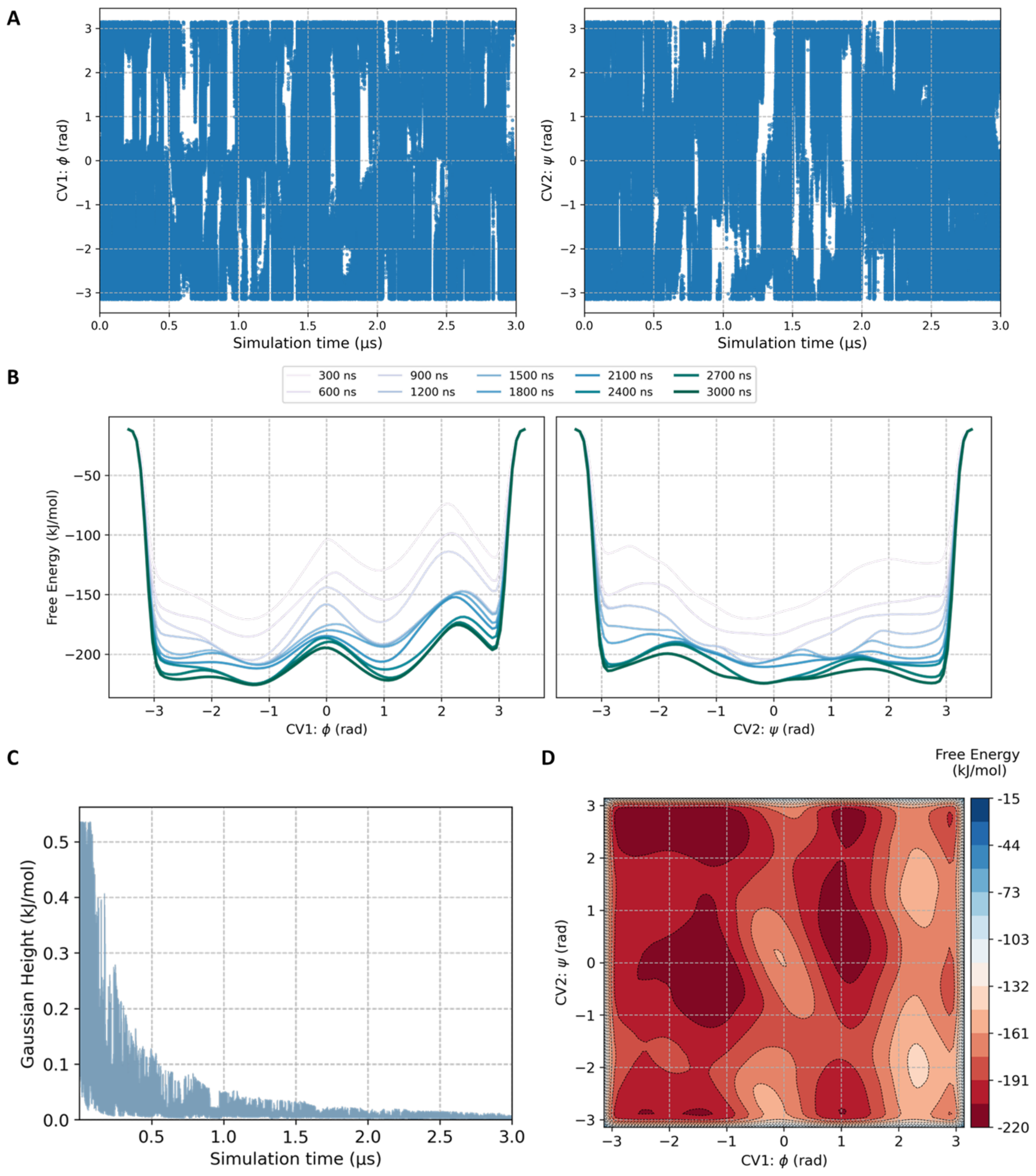

Figure S8. Metadynamics simulation results for E219A variants. (A) Time evolution of the two collective variables (CVs) over the entire 3 μs metadynamics run, showing CV_1 (ϕ, in radians; left) and CV_2 (ψ, in radians; right) as functions of simulation time. (B) One-dimensional free-energy profiles extracted at cumulative metadynamics times of 300–3000 ns for CV_1 (left) and CV_2 (right). (C) Height of added Gaussian bias potentials as a function of simulation time. The Gaussian height decays rapidly from approximately 0.5 kJ/mol toward near zero by ~3 μs. (D) Final two-dimensional free-energy surface (FES) reconstructed from metadynamics, plotted with CV_1 (ϕ, in radians) on the horizontal axis and CV_2 (ψ, in radians) on the vertical axis. The color scale (right) denotes relative free energy in kJ/mol: low-energy basins appear in dark shades, whereas high-energy regions and barriers appear in lighter shades.

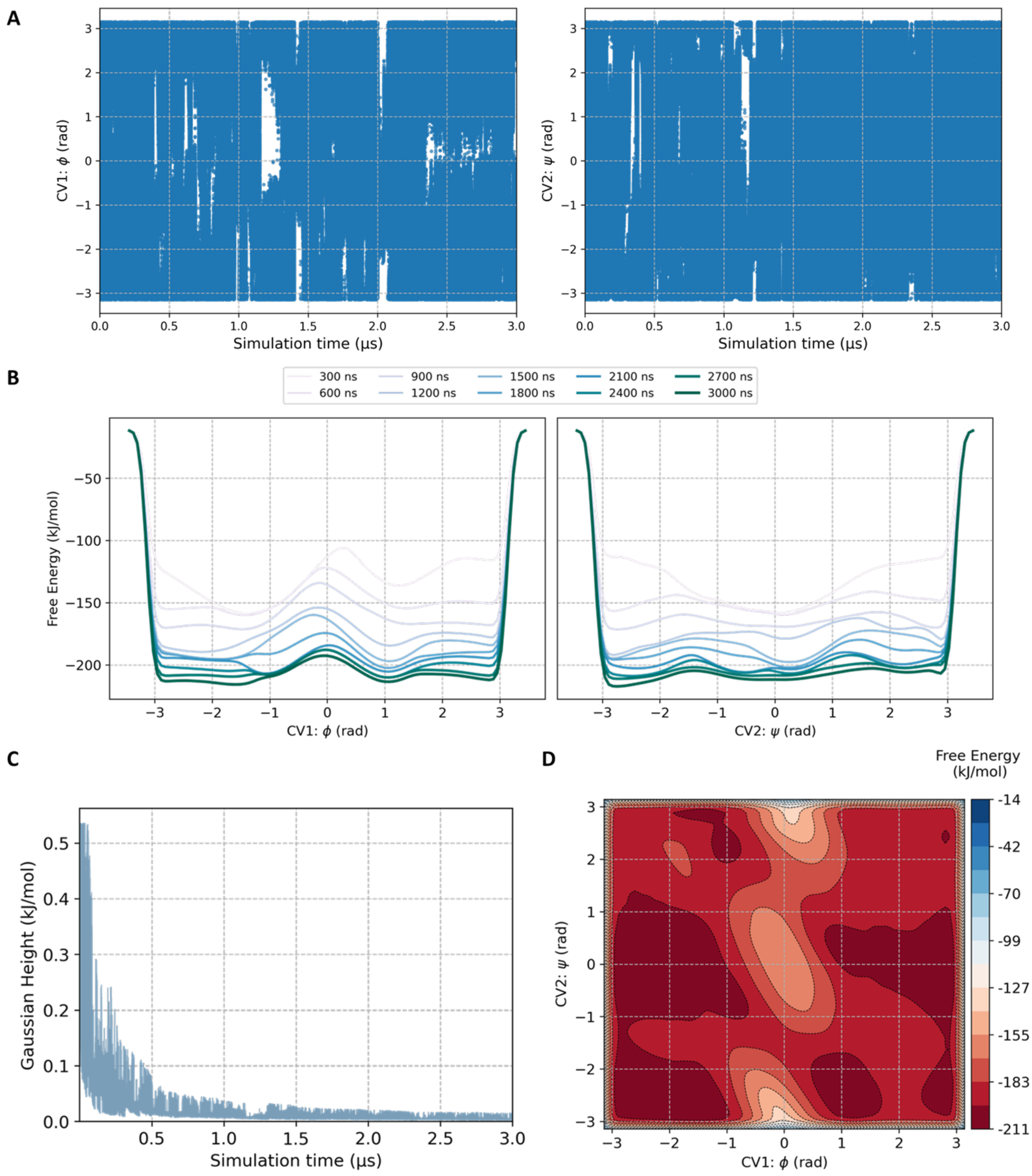

Figure S9. Metadynamics simulation results for E219G variants. (A) Time evolution of the two collective variables (CVs) over the entire 3 μs metadynamics run, showing CV_1 (ϕ, in radians; left) and CV_2 (ψ, in radians; right) as functions of simulation time. (B) One-dimensional free-energy profiles extracted at cumulative metadynamics times of 300–3000 ns for CV_1 (left) and CV_2 (right). (C) Height of added Gaussian bias potentials as a function of simulation time. The Gaussian height decays rapidly from approximately 0.5 kJ/mol toward near zero by ~3 μs. (D) Final two-dimensional free-energy surface (FES) reconstructed from metadynamics, plotted with CV_1 (ϕ, in radians) on the horizontal axis and CV_2 (ψ, in radians) on the vertical axis. The color scale (right) denotes relative free energy in kJ/mol: low-energy basins appear in dark shades, whereas high-energy regions and barriers appear in lighter shades.

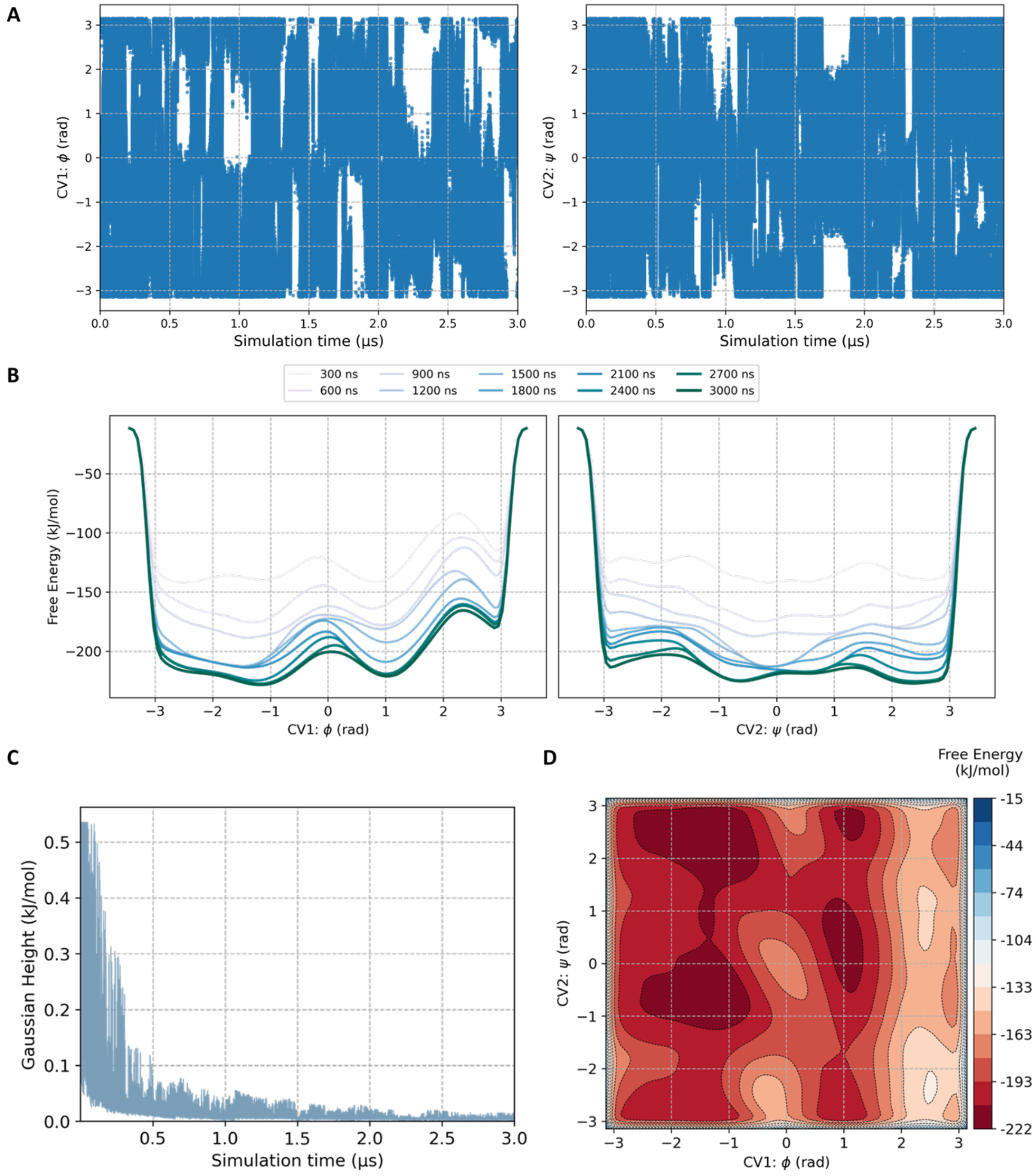

Figure S10. Metadynamics simulation results for E219K variants. (A) Time evolution of the two collective variables (CVs) over the entire 3 μs metadynamics run, showing CV_1 (ϕ, in radians; left) and CV_2 (ψ, in radians; right) as functions of simulation time. (B) One-dimensional free-energy profiles extracted at cumulative metadynamics times of 300–3000 ns for CV_1 (left) and CV_2 (right). (C) Height of added Gaussian bias potentials as a function of simulation time. The Gaussian height decays rapidly from approximately 0.5 kJ/mol toward near zero by ~3 μs. (D) Final two-dimensional free-energy surface (FES) reconstructed from metadynamics, plotted with CV_1 (ϕ, in radians) on the horizontal axis and CV_2 (ψ, in radians) on the vertical axis. The color scale (right) denotes relative free energy in kJ/mol: low-energy basins appear in dark shades, whereas high-energy regions and barriers appear in lighter shades.

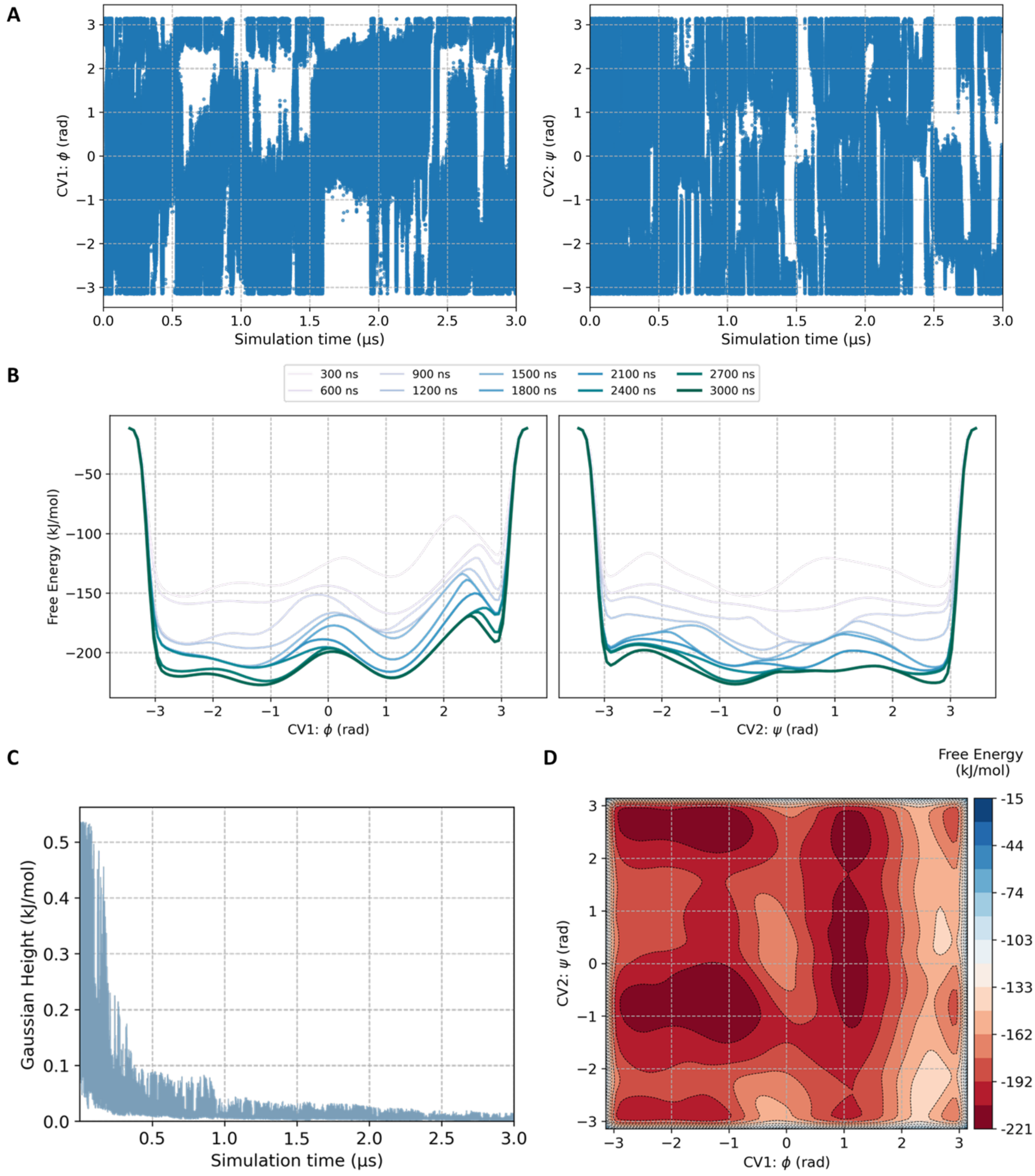

Figure S11. Metadynamics simulation results for wild-type PDC-3 (Y221 system). (A) Time evolution of the two collective variables (CVs) over the entire 3 μs metadynamics run, showing CV_1 (ϕ, in radians; left) and CV_2 (ψ, in radians; right) as functions of simulation time. (B) One-dimensional free-energy profiles extracted at cumulative metadynamics times of 300–3000 ns for CV_1 (left) and CV_2 (right). (C) Height of added Gaussian bias potentials as a function of simulation time. The Gaussian height decays rapidly from approximately 0.5 kJ/mol toward near zero by ~3 μs. (D) Final two-dimensional free-energy surface (FES) reconstructed from metadynamics, plotted with CV_1 (ϕ, in radians) on the horizontal axis and CV_2 (ψ, in radians) on the vertical axis. The color scale (right) denotes relative free energy in kJ/mol: low-energy basins appear in dark shades, whereas high-energy regions and barriers appear in lighter shades.

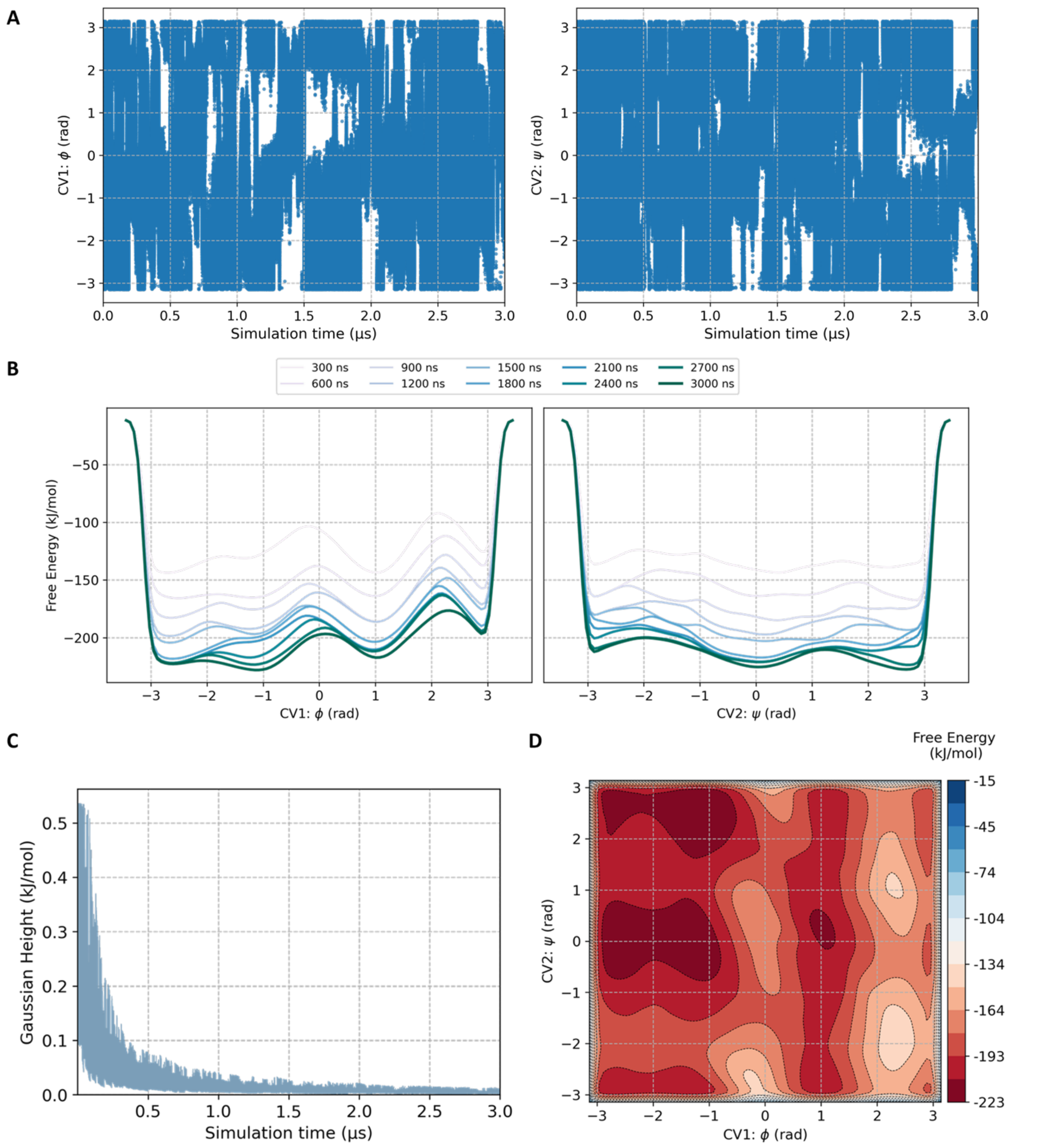

Figure S12. Metadynamics simulation results for Y221A variants. (A) Time evolution of the two collective variables (CVs) over the entire 3 μs metadynamics run, showing CV_1 (ϕ, in radians; left) and CV_2 (ψ, in radians; right) as functions of simulation time. (B) One-dimensional free-energy profiles extracted at cumulative metadynamics times of 300–3000 ns for CV_1 (left) and CV_2 (right). (C) Height of added Gaussian bias potentials as a function of simulation time. The Gaussian height decays rapidly from approximately 0.5 kJ/mol toward near zero by ~3 μs. (D) Final two-dimensional free-energy surface (FES) reconstructed from metadynamics, plotted with CV_1 (ϕ, in radians) on the horizontal axis and CV_2 (ψ, in radians) on the vertical axis. The color scale (right) denotes relative free energy in kJ/mol: low-energy basins appear in dark shades, whereas high-energy regions and barriers appear in lighter shades.

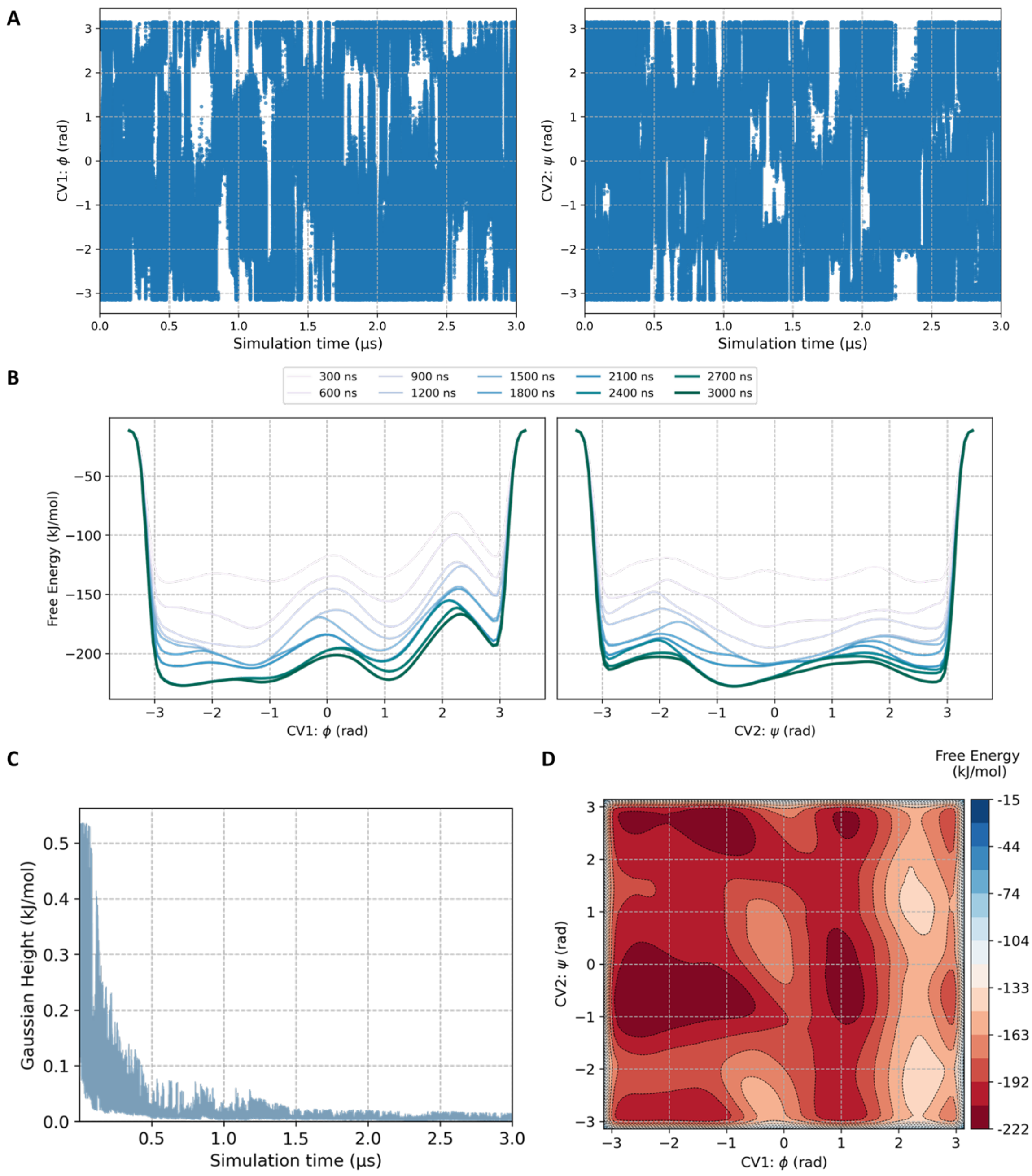

Figure S13. Metadynamics simulation results for Y221H variants. (A) Time evolution of the two collective variables (CVs) over the entire 3 μs metadynamics run, showing CV_1 (ϕ, in radians; left) and CV_2 (ψ, in radians; right) as functions of simulation time. (B) One-dimensional free-energy profiles extracted at cumulative metadynamics times of 300–3000 ns for CV_1 (left) and CV_2 (right). (C) Height of added Gaussian bias potentials as a function of simulation time. The Gaussian height decays rapidly from approximately 0.5 kJ/mol toward near zero by ~3 μs. (D) Final two-dimensional free-energy surface (FES) reconstructed from metadynamics, plotted with CV_1 (ϕ, in radians) on the horizontal axis and CV_2 (ψ, in radians) on the vertical axis. The color scale (right) denotes relative free energy in kJ/mol: low-energy basins appear in dark shades, whereas high-energy regions and barriers appear in lighter shades.

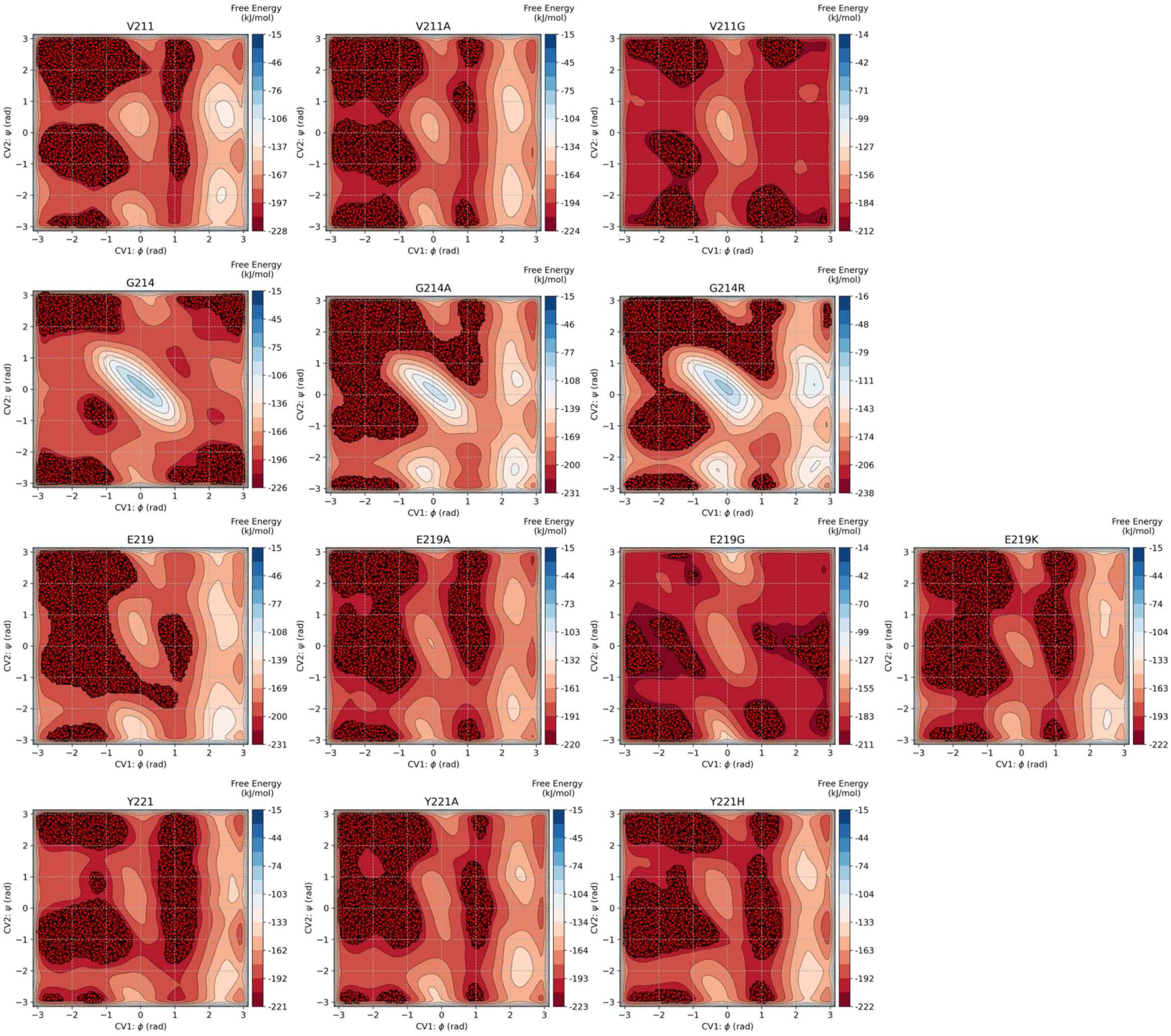

Figure S14. Free-energy landscapes for WT-MetaD simulations biasing individual Ω-loop residues. Each panel shows the reconstructed free energy (in kJ/mol) as a function of the ϕ (horizontal) and ψ (vertical) dihedrals of a single residue, with panels arranged in four rows corresponding to residues 211 (wild-type PDC-3, V211A, and V211G variants), 214 (wild-type PDC-3, G214A, and G214R variants), 219 (wild-type PDC-3, E219A, E219G, and E219K), and 221 (wild-type PDC-3, Y221A, and Y221H). In every free-energy landscape, red dots mark conformations whose free energy falls below −200 kJ/mol.

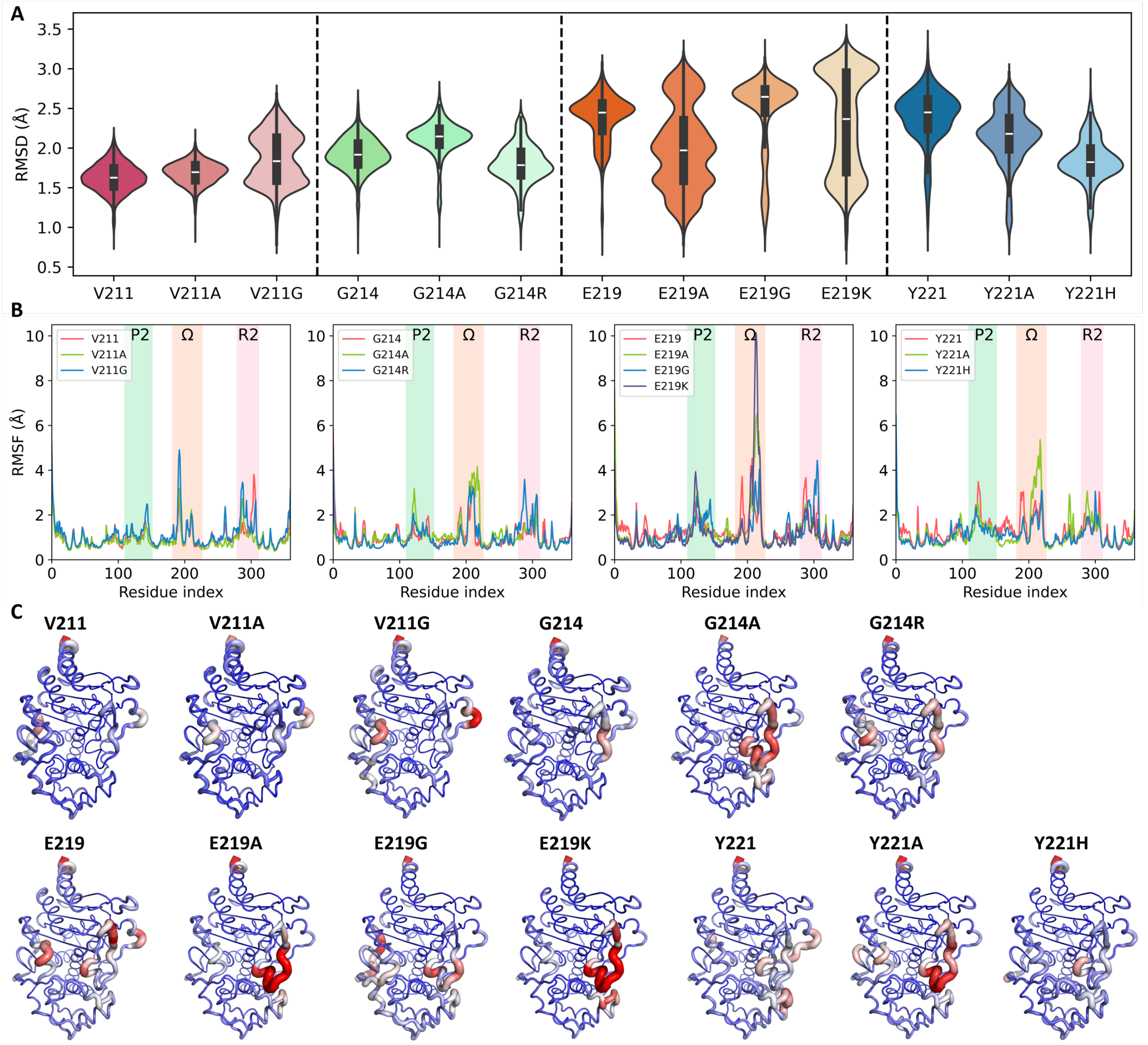

Figure S15. Global and local conformational changes induced by backbone-torsion biasing at residues 211, 214, 219, and 221 of wild-type and mutant PDC-3. (A) Violin plots of RMSD distributions (in Å) for low-free-energy frames (free energy < -200 kJ/mol) obtained when backbone ψ/ϕ torsions at residues 211, 214, 219, and 221 are biased. The white line indicates the median, and the black box denotes the interquartile range. (B) Per-residue RMSF profiles (in Å) for each torsion-biasing condition. Shaded regions mark the P2-, the Ω-, and the R2-loops. (C) Ribbon representations of PDC-3 under each torsion bias, colored from blue→white→red according to per-residue RMSF and displayed with tube thickness proportional to RMSF magnitude.

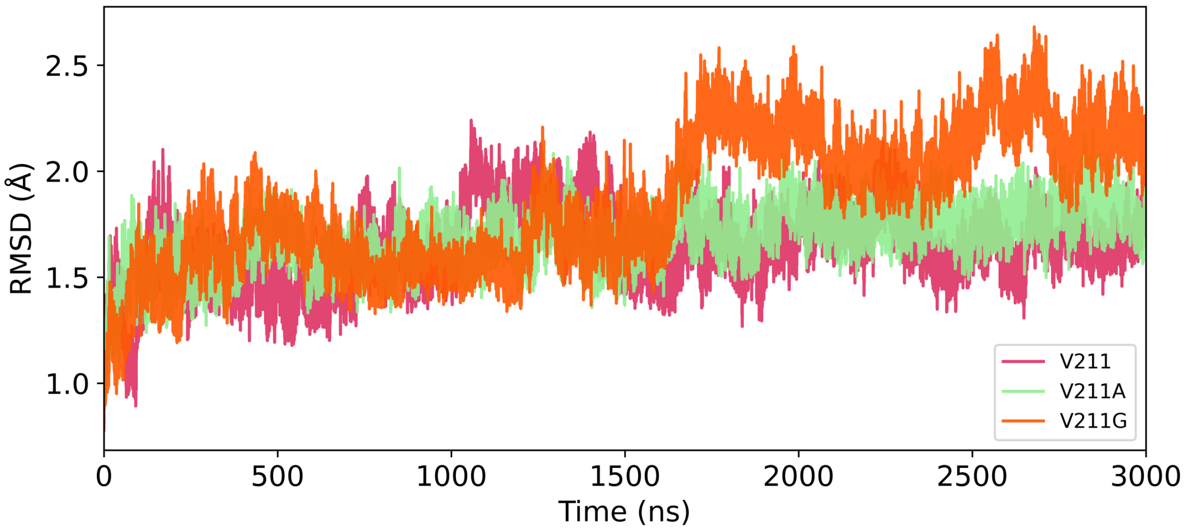

Figure S16. Time evolution of Cα RMSD for wild-type PDC-3 and variants under backbone ψ/ϕ bias at residue 211.

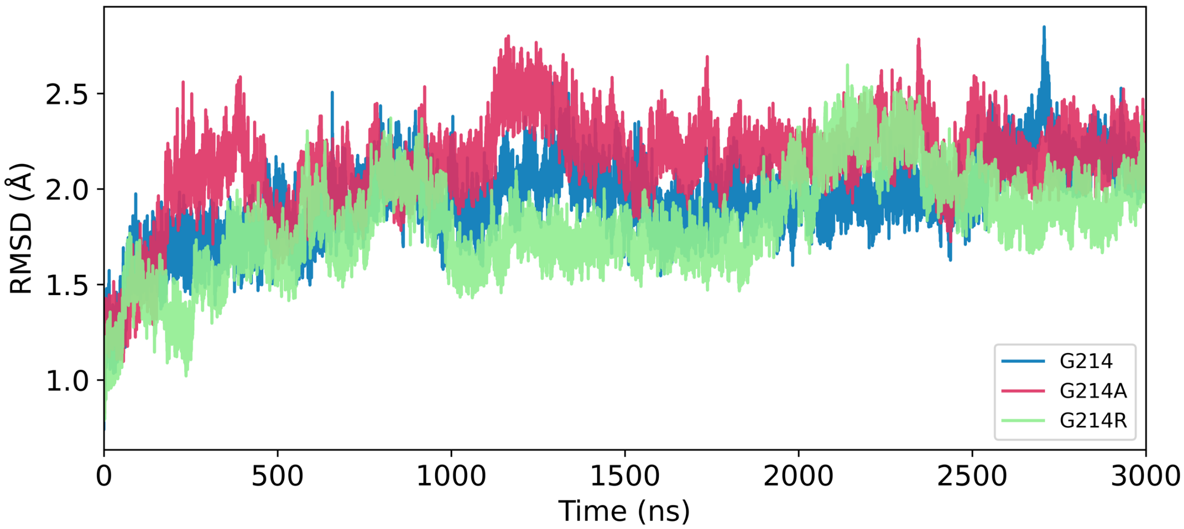

Figure S17. Time evolution of Cα RMSD for wild-type PDC-3 and variants under backbone ψ/ϕ bias at residue 214.

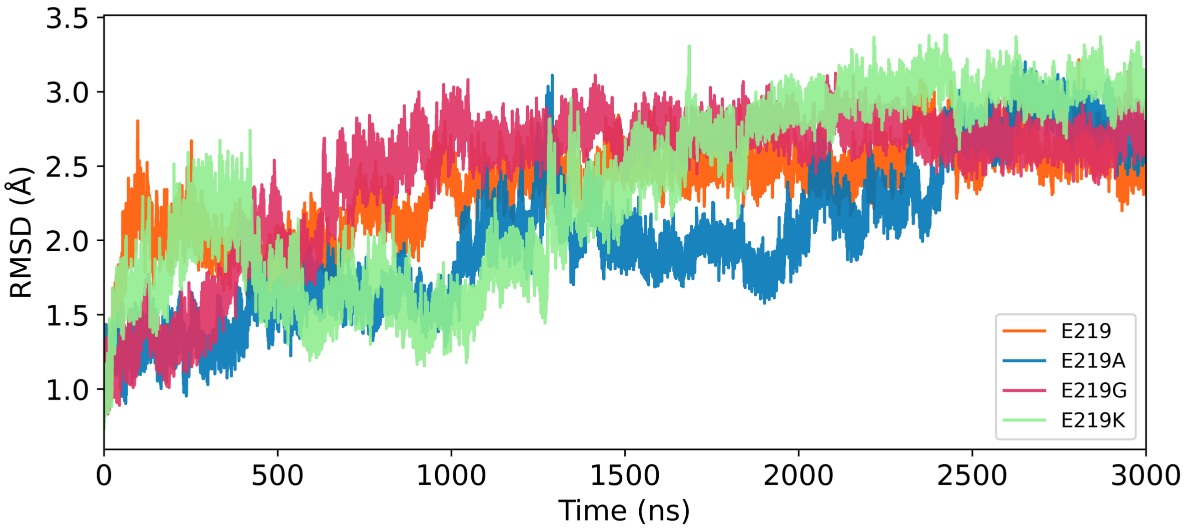

Figure S18. Time evolution of Cα RMSD for wild-type PDC-3 and variants under backbone ψ/ϕ bias at residue 219.

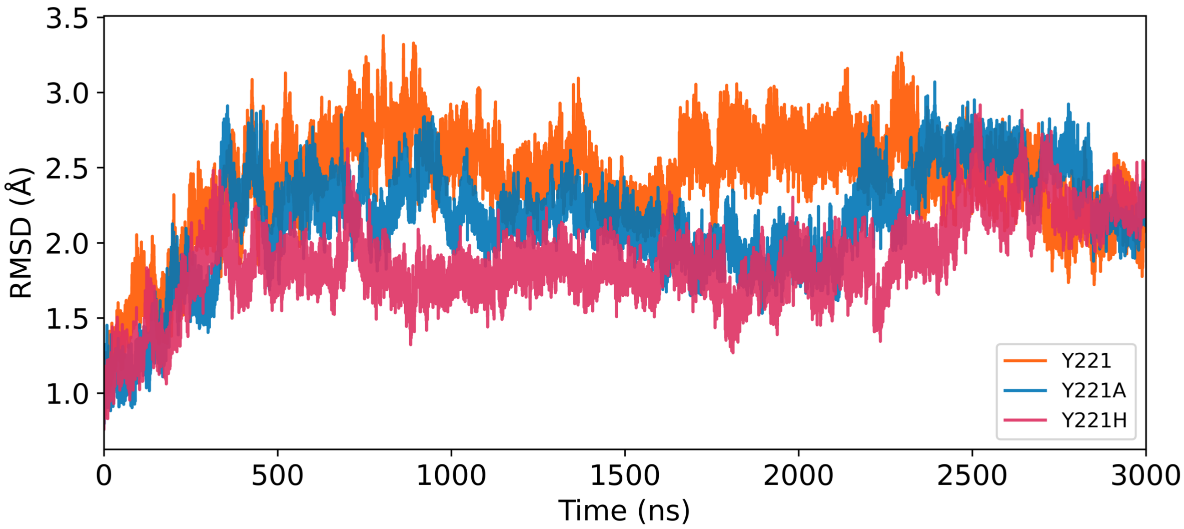

Figure S19. Time evolution of Cα RMSD for wild-type PDC-3 and variants under backbone ψ/ϕ bias at residue 221.

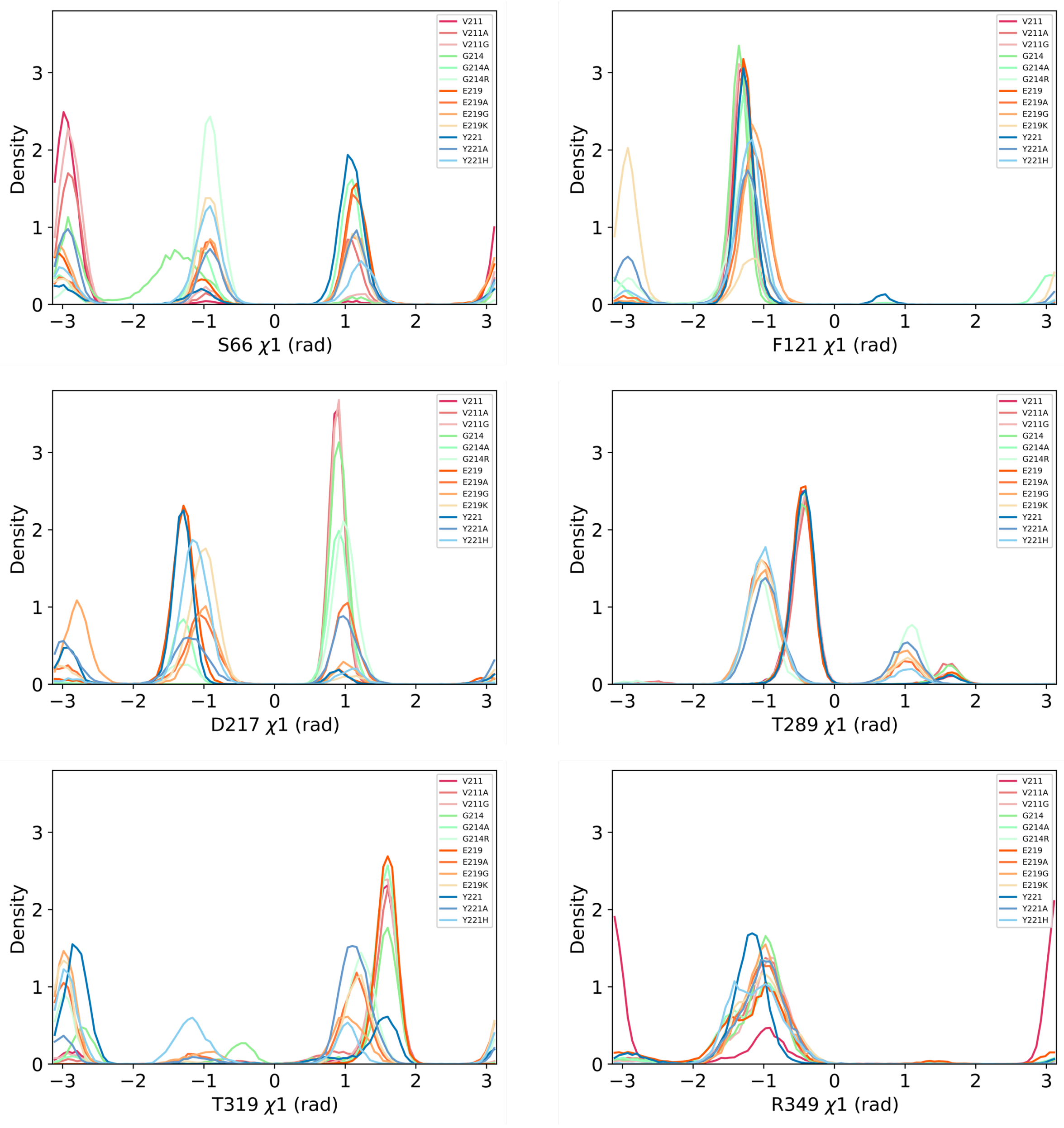

Figure S20. Sidechain χ_1 torsion-angle distributions for six active-site residues across all systems. For each residue (S66, F121, D217, T289, T319, R349), we computed a normalized histogram over 100 bins spanning $[-\pi, \pi]$, then applied a Savitzky-Golay filter to smooth the counts. The resulting smoothed densities (y-axis) are plotted against χ_1 (radians, x-axis).

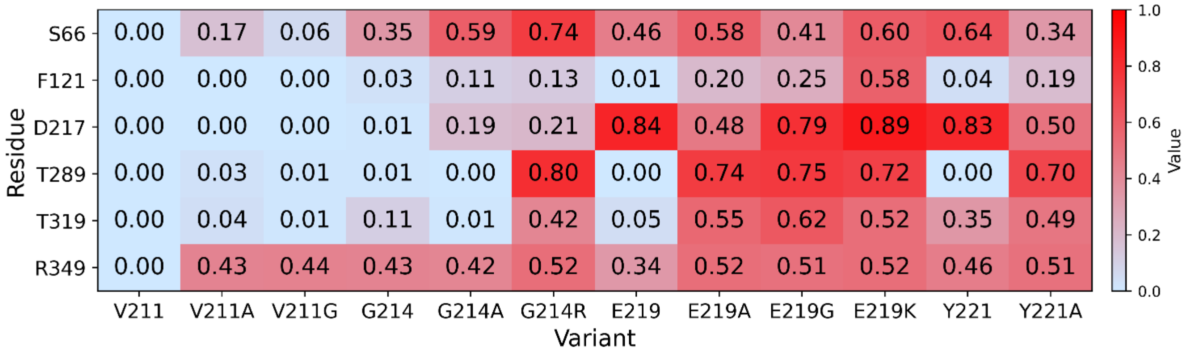

Figure S14. Jensen-Shannon (JS) divergence of sidechain χ1 distributions for six active-site residues, referenced to the wild-type PDC-3 (V211 system).

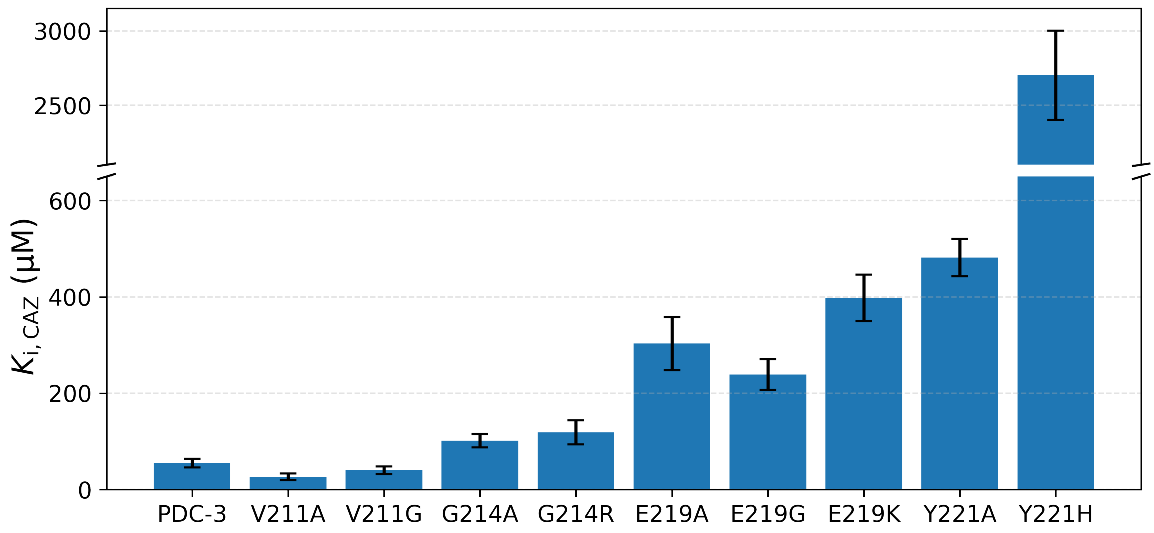

Figure S15. The apparent competitive inhibition constants for ceftazidime for wild-type PDC-3 and its variants.

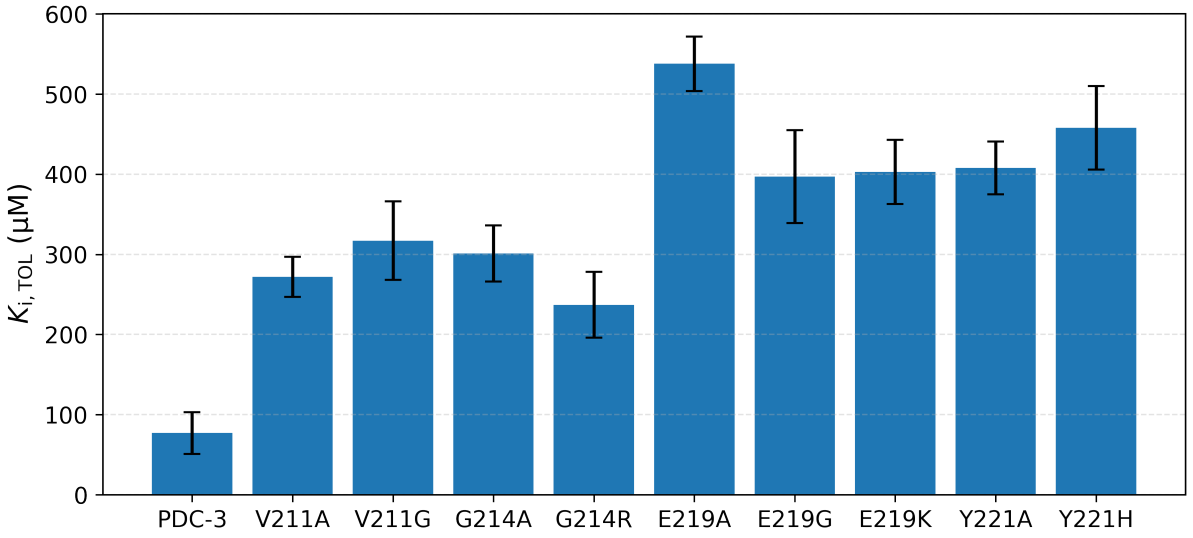

Figure S16. The apparent competitive inhibition constants for ceftolozane for wild-type PDC-3 and its variants.

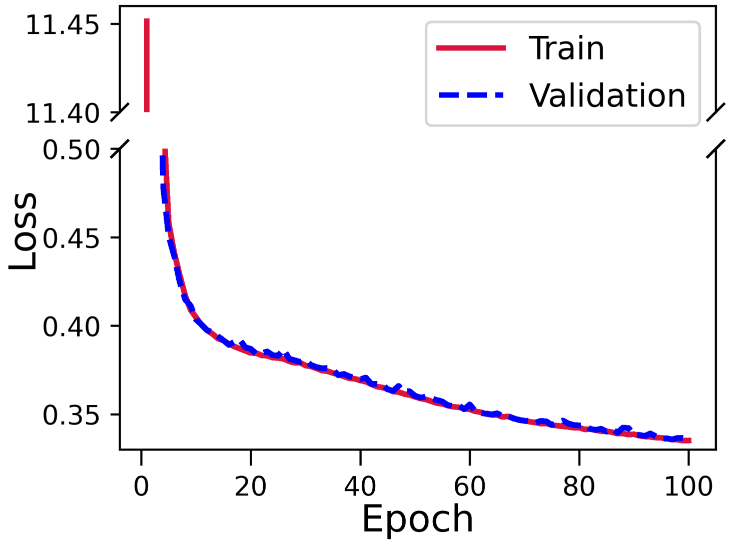

Figure S17. CVAE training and validation loss convergence over 100 epochs. The plot shows the total VAE loss (red solid line for training, blue dashed line for validation) as a function of epoch on a logarithmic scale. Both curves drop precipitously during the first few epochs, then gradually converge to a low and stable value, indicating that the model successfully learned to reconstruct the 53×53 distance matrices while satisfying the KL divergence constraint. The close overlap of training and validation losses confirms that the CVAE did not overfit and that the 8-dimensional latent space provides a robust representation of the Ω-loop conformational ensemble.

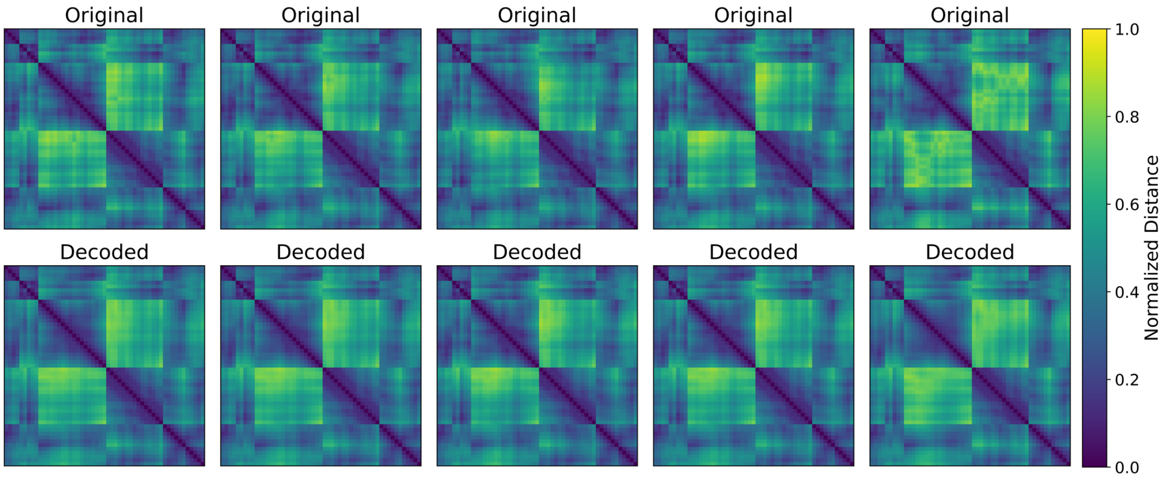

Figure S18. Original versus CVAE-reconstructed distance matrices for five randomly chosen frames. The top row (labelled ‘Original’) shows five distinct 53 × 53 Cα distance matrices extracted from simulation snapshots across all WT-MetaD runs. Each matrix encodes pairwise distances among conserved catalytic motifs, the Ω-loop, helix 10, and some critical active-site residues (L119, Q120, N246, N343, P345, R349). The corresponding CVAE-decoded matrices (bottom row, labelled ‘Decoded’) closely reproduce these distance patterns, demonstrating that the trained model can accurately reconstruct the detailed contact maps of diverse Ω-loop conformations. Color intensity reflects normalized Cα-Cα distances (0.0 = purple to 1.0 = yellow).

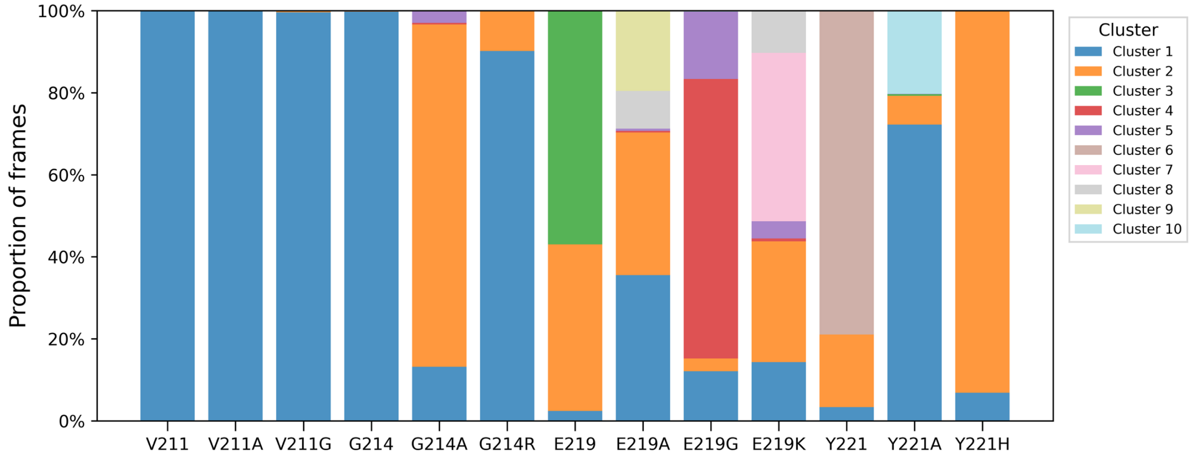

Figure S19. Proportion of simulation frames assigned to each of the 10 clusters for all systems. For each system, the proportion of frames assigned to each cluster is shown as a stacked bar, obtained from all frames with free energy < –200 kJ·mol^{-1} across the corresponding WT-MetaD trajectory.

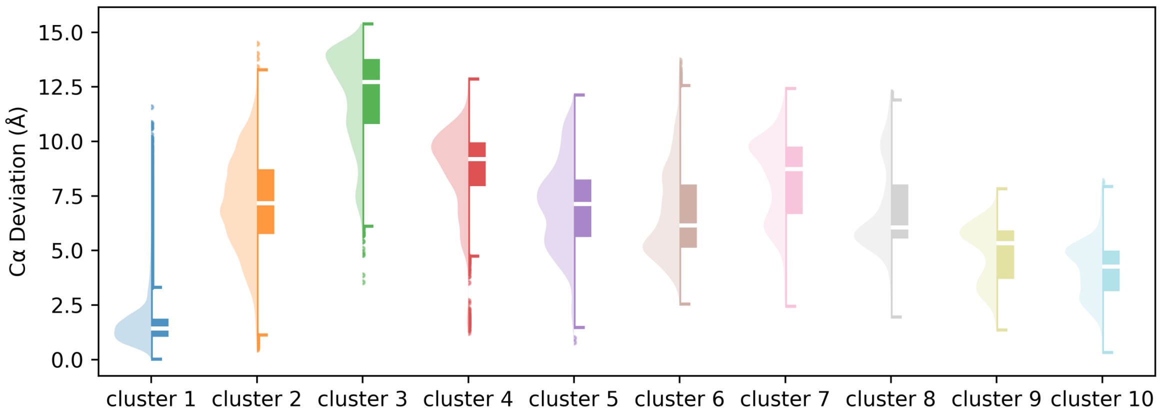

Figure S20. Distribution of G220 Cα displacements from the crystal reference across all clusters. After least-squares alignment of each low-energy frame to the wild-type PDC-3 crystal structure, the Cα position of residue G220 was compared to its crystallographic coordinate. Violin-box plots show the resulting G220 displacements for clusters 1-10. Each plot shows the full distribution (violin), median and interquartile range (box), whiskers at 1.5× IQR, and outliers as individual points.

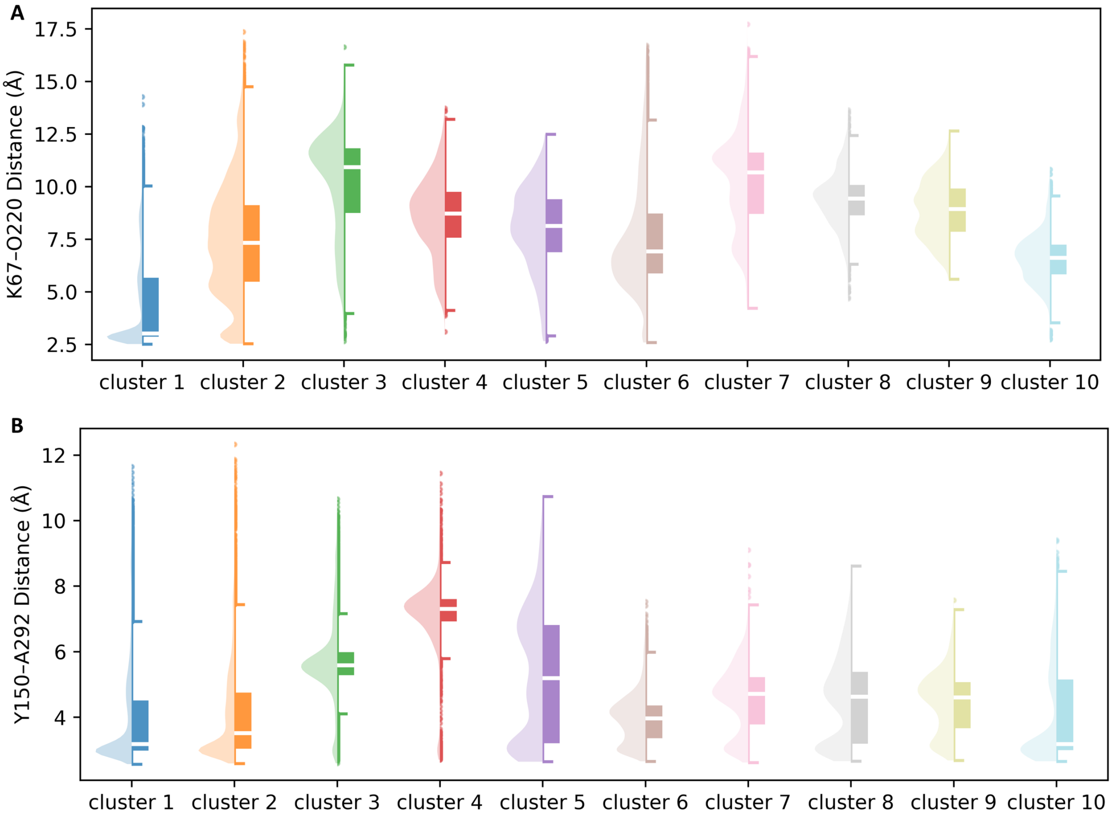

Figure S21. Distribution of donor-acceptor distances for hydrogen bonds K67-G220 and Y150-A292 which regulate active-site expansion and constriction across all clusters. Violin-box plots show the (A) K67-G220 and the (B) Y150-A292 donor-acceptor distance for clusters 1-10. Each plot shows the full distribution (violin), median and interquartile range (box), whiskers at 1.5× IQR, and outliers as individual points.

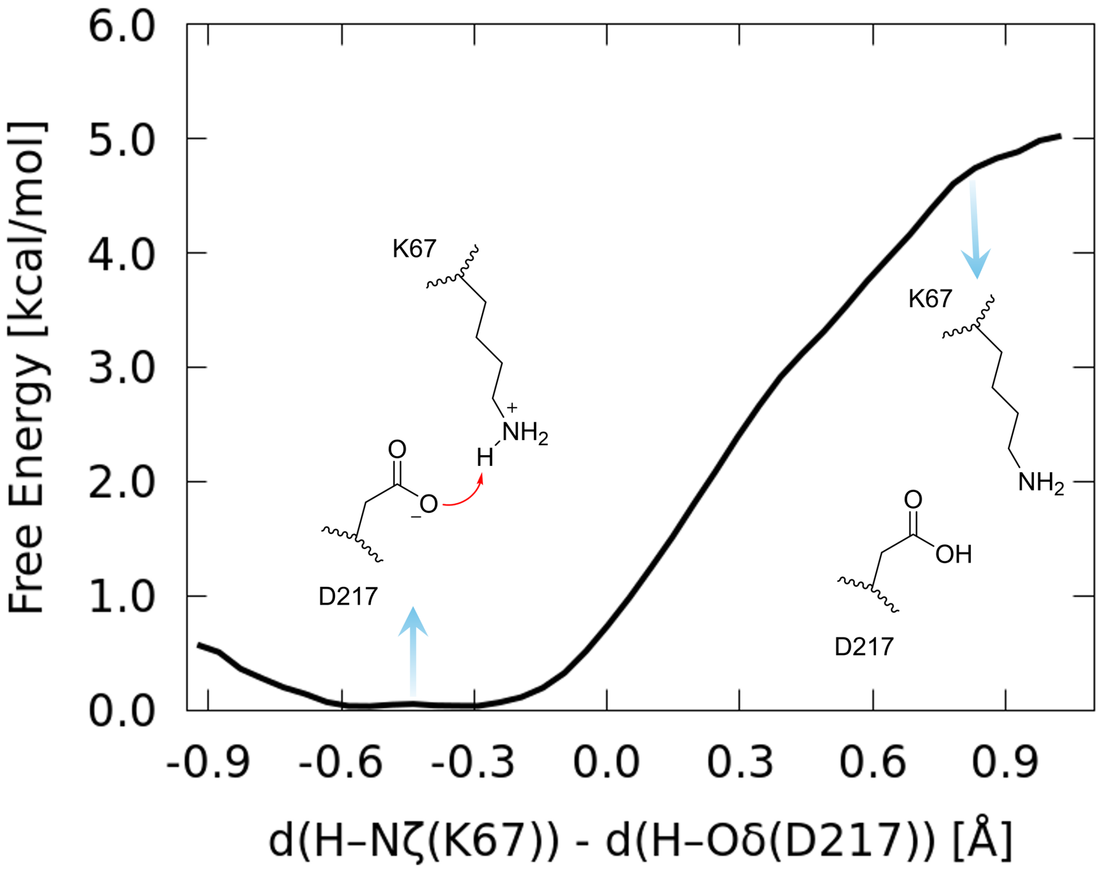

Figure S22. Free energy profile for the direct proton transfer from K67 to D217.

Tables

Table S1. Frame counts per system in WT-MetaD simulations. The WT-MetaD Frames column shows the total number of frames collected for each system, while the Low-energy Frames column lists the number of frames exhibiting bias-corrected free energies below −200 kJ/mol.

	WT-MetaD Frames	Low-energy Frames
V211	30000	12964
V211A	30000	12821
V211G	30000	7134
G214	30000	8750
G214A	30000	15491
G214R	30000	15581
E219	30000	14717
E219A	30000	13365
E219G	30000	6364
E219K	30000	14303
Y221	30000	12598
Y221A	30000	12518
Y221H	30000	12816
Total	390000	159422

Table S2. Jensen-Shannon (JS) divergence of sidechain χ_1 distributions, referenced to the wild-type PDC-3 (V211 system). The table lists the JS divergence between each variant (columns) and wild-type PDC-3 with backbone bias at V211 (reference). A value of 0 denotes unchanged rotamer preferences, whereas values approaching 1 indicate a substantial redistribution of χ_1 populations. Divergences equal to or exceeding 0.50 are taken as the threshold for a significant conformational shift.

	V211	V211A	V211G	G214	G214A	G214R	E219	E219A	E219G	E219K	Y221	Y221A	Y221H
**D6**	0.00	0.10	0.03	0.02	0.32	0.41	0.00	0.31	0.50	0.43	0.00	0.34	0.25
**V16**	0.00	0.18	0.03	0.03	0.03	0.55	0.00	0.74	0.63	0.47	0.11	0.43	0.77
**D24**	0.00	0.00	0.00	0.01	0.14	0.53	0.04	0.50	0.49	0.48	0.01	0.51	0.53
**Y40**	0.00	0.01	0.00	0.11	0.00	0.69	0.00	0.47	0.61	0.69	0.02	0.69	0.64
**E61**	0.00	0.01	0.02	0.01	0.01	0.06	0.01	0.12	0.10	0.06	0.10	0.09	0.70
**S66**	0.00	0.17	0.06	0.35	0.59	0.74	0.46	0.58	0.41	0.60	0.64	0.34	0.52
**T68**	0.00	0.24	0.07	0.41	0.15	0.48	0.30	0.50	0.54	0.44	0.26	0.52	0.60
**T70**	0.00	0.25	0.08	0.26	0.09	0.64	0.04	0.59	0.66	0.67	0.16	0.65	0.54
**D81**	0.00	0.00	0.01	0.00	0.00	0.56	0.00	0.56	0.57	0.56	0.01	0.57	0.58
**D86**	0.00	0.36	0.43	0.51	0.41	0.56	0.29	0.49	0.54	0.53	0.51	0.45	0.44
**D87**	0.00	0.05	0.16	0.24	0.11	0.52	0.08	0.39	0.40	0.44	0.40	0.30	0.36
**F101**	0.00	0.00	0.09	0.45	0.00	0.27	0.01	0.55	0.23	0.29	0.01	0.47	0.39
**D102**	0.00	0.04	0.09	0.48	0.06	0.30	0.01	0.55	0.22	0.26	0.09	0.47	0.33
**T111**	0.00	0.41	0.48	0.50	0.46	0.68	0.18	0.61	0.67	0.61	0.41	0.69	0.63
**Y112**	0.00	0.62	0.02	0.02	0.03	0.86	0.83	0.85	0.86	0.86	0.40	0.51	0.83
**T113**	0.00	0.11	0.02	0.21	0.07	0.61	0.09	0.60	0.66	0.57	0.06	0.61	0.60
**F121**	0.00	0.00	0.00	0.03	0.11	0.13	0.01	0.20	0.25	0.58	0.04	0.19	0.13
**I155**	0.00	0.20	0.04	0.27	0.08	0.17	0.51	0.15	0.40	0.87	0.39	0.21	0.44
**F170**	0.00	0.01	0.00	0.00	0.03	0.52	0.06	0.29	0.19	0.26	0.03	0.38	0.34
**F179**	0.00	0.70	0.32	0.03	0.14	0.78	0.07	0.78	0.76	0.77	0.07	0.73	0.49
**Y199**	0.00	0.00	0.00	0.00	0.00	0.36	0.01	0.46	0.45	0.44	0.02	0.37	0.56
**D206**	0.00	0.00	0.00	0.01	0.11	0.40	0.03	0.50	0.48	0.51	0.01	0.37	0.51
**D217**	0.00	0.00	0.00	0.01	0.19	0.21	0.84	0.48	0.79	0.89	0.83	0.50	0.85
**H239**	0.00	0.02	0.01	0.44	0.03	0.23	0.70	0.05	0.13	0.31	0.02	0.11	0.18
**T255**	0.00	0.04	0.17	0.15	0.15	0.64	0.16	0.61	0.69	0.55	0.03	0.62	0.59
**H256**	0.00	0.49	0.38	0.31	0.49	0.44	0.22	0.32	0.32	0.42	0.44	0.60	0.33
**Y260**	0.00	0.67	0.51	0.64	0.64	0.78	0.66	0.74	0.27	0.76	0.63	0.49	0.70
**D264**	0.00	0.48	0.09	0.06	0.20	0.53	0.07	0.58	0.38	0.57	0.17	0.30	0.50
**T266**	0.00	0.09	0.13	0.06	0.19	0.51	0.14	0.51	0.54	0.54	0.15	0.45	0.54
**Q267**	0.00	0.09	0.02	0.04	0.03	0.30	0.07	0.14	0.65	0.10	0.01	0.22	0.12
**D275**	0.00	0.54	0.33	0.06	0.49	0.52	0.11	0.54	0.33	0.54	0.52	0.55	0.54
**T289**	0.00	0.03	0.01	0.01	0.00	0.80	0.00	0.74	0.75	0.72	0.00	0.70	0.72
**I298**	0.00	0.03	0.37	0.72	0.64	0.37	0.06	0.23	0.60	0.25	0.16	0.64	0.13
**R300**	0.00	0.06	0.13	0.62	0.54	0.41	0.09	0.34	0.45	0.26	0.19	0.69	0.29
**T319**	0.00	0.04	0.01	0.11	0.01	0.42	0.05	0.55	0.62	0.52	0.35	0.49	0.73
**D333**	0.00	0.03	0.03	0.05	0.00	0.52	0.01	0.35	0.47	0.67	0.00	0.39	0.52
**I338**	0.00	0.15	0.14	0.10	0.12	0.42	0.05	0.51	0.38	0.40	0.36	0.45	0.51
**Y344**	0.00	0.01	0.00	0.03	0.00	0.10	0.01	0.16	0.06	0.08	0.53	0.03	0.17
**R349**	0.00	0.43	0.44	0.43	0.42	0.52	0.34	0.52	0.51	0.52	0.46	0.51	0.52
**V350**	0.00	0.49	0.33	0.38	0.03	0.35	0.30	0.48	0.49	0.49	0.20	0.51	0.45

Table S3. Number of simulation frames assigned to each of the 10 clusters for all systems.

	1	2	3	4	5	6	7	8	9	10
**V211**	12950	14	0	0	0	0	0	0	0	0
**V211A**	12801	20	0	0	0	0	0	0	0	0
**V211G**	7106	28	0	0	0	0	0	0	0	0
**G214**	8726	20	0	0	0	0	0	0	0	4
**G214A**	2043	12931	0	62	454	0	0	0	0	1
**G214R**	14047	1525	9	0	0	0	0	0	0	0
**E219**	356	5978	8383	0	0	0	0	0	0	0
**E219A**	4750	4644	0	52	72	1	0	1230	2601	15
**E219G**	771	196	0	4337	1044	0	0	0	0	16
**E219K**	2050	4209	0	102	598	0	5874	1468	0	2
**Y221**	424	2224	0	0	0	9950	0	0	0	0
**Y221A**	9044	874	54	0	9	0	0	0	0	2537
**Y221H**	882	11924	3	0	1	5	0	1	0	0

Table S4. Proportion of simulation frames assigned to each of the 10 clusters for all systems.

	1	2	3	4	5	6	7	8	9	10
**V211**	99.89%	0.11%	0.00%	0.00%	0.00%	0.00%	0.00%	0.00%	0.00%	0.00%
**V211A**	99.84%	0.16%	0.00%	0.00%	0.00%	0.00%	0.00%	0.00%	0.00%	0.00%
**V211G**	99.61%	0.39%	0.00%	0.00%	0.00%	0.00%	0.00%	0.00%	0.00%	0.00%
**G214**	99.73%	0.23%	0.00%	0.00%	0.00%	0.00%	0.00%	0.00%	0.00%	0.05%
**G214A**	13.19%	83.47%	0.00%	0.40%	2.93%	0.00%	0.00%	0.00%	0.00%	0.01%
**G214R**	90.15%	9.79%	0.06%	0.00%	0.00%	0.00%	0.00%	0.00%	0.00%	0.00%
**E219**	2.42%	40.62%	56.96%	0.00%	0.00%	0.00%	0.00%	0.00%	0.00%	0.00%
**E219A**	35.54%	34.75%	0.00%	0.39%	0.54%	0.01%	0.00%	9.20%	19.46%	0.11%
**E219G**	12.12%	3.08%	0.00%	68.15%	16.40%	0.00%	0.00%	0.00%	0.00%	0.25%
**E219K**	14.33%	29.43%	0.00%	0.71%	4.18%	0.00%	41.07%	10.26%	0.00%	0.01%
**Y221**	3.37%	17.65%	0.00%	0.00%	0.00%	78.98%	0.00%	0.00%	0.00%	0.00%
**Y221A**	72.25%	6.98%	0.43%	0.00%	0.07%	0.00%	0.00%	0.00%	0.00%	20.27%
**Y221H**	6.88%	93.04%	0.02%	0.00%	0.01%	0.04%	0.00%	0.01%	0.00%	0.00%

Table S5. Quantitative characterization of conformational differences across clusters 1–10. G220 Cα deviations reflect the displacement (Å) from the wild-type crystal structure, indicating Ω-loop movement. Distances (Å) of K67–G220 and Y150–A292 hydrogen bonds represent disruption of key interactions stabilizing the active site. Values shown as mean ± SD.

cluster	G220 C_α Deviation	K67-G220 Distance	Y150-A292 Distance
**1**	1.59±0.94	4.38±2.08	3.84±1.34
**2**	7.18±2.12	7.40±2.63	4.03±1.31
**3**	12.08±2.13	10.16±2.31	5.69±1.22
**4**	8.75±1.78	8.59±1.80	7.16±1.01
**5**	7.07±1.97	8.00±1.92	5.26±1.91
**6**	6.62±1.98	7.53±2.52	3.93±0.73
**7**	8.29±1.86	10.25±2.18	4.57±1.01
**8**	6.86±1.92	9.38±1.30	4.51±1.27
**9**	4.89±1.33	8.89±1.34	4.45±0.93
**10**	4.11±1.22	6.58±1.12	4.03±1.44
